# Coding Dynamics of the Striatal Networks During Learning

**DOI:** 10.1523/ENEURO.0436-23.2024

**Published:** 2024-10-23

**Authors:** Maxime Villet, Patricia Reynaud-Bouret, Julien Poitreau, Jacopo Baldi, Sophie Jaffard, Ashwin James, Alexandre Muzy, Evgenia Kartsaki, Gilles Scarella, Francesca Sargolini, Ingrid Bethus

**Affiliations:** ^1^Université Côte d’Azur, CNRS, INSERM, Institut de Pharmacologie Moléculaire et Cellulaire, Valbonne 06560, France; ^2^Université Côte d’Azur, CNRS, LJAD and NeuroMod, Nice 0600, France; ^3^CRPN, UMR 7077, Aix-Marseille University, CNRS, Marseille 13331, France; ^4^Université Côte d’Azur, CNRS, I3S, Valbonne 06560, France; ^5^Université Côte d’Azur, NeuroMod, Valbonne 06560, France

## Abstract

The rat dorsomedial (DMS) and dorsolateral striatum (DLS), equivalent to caudate nucleus and putamen in primates, are required for goal-directed and habit behaviour, respectively. However, it is still unclear whether and how this functional dichotomy emerges in the course of learning. In this study, we investigated this issue by recording DMS and DLS single neuron activity in rats performing a continuous spatial alternation task, from the acquisition to optimized performance. We first applied a classical analytical approach to identify task-related activity based on the modifications of single neuron firing rate in relation to specific task events or maze trajectories. We then used an innovative approach based on Hawkes process to reconstruct a directed connectivity graph of simultaneously recorded neurons, that was used to decode animal behavior. This approach enabled us to better unravel the role of DMS and DLS neural networks across learning stages. We showed that DMS and DLS display different task-related activity throughout learning stages, and the proportion of coding neurons over time decreases in the DMS and increases in the DLS. Despite these major differences, the decoding power of both networks increases during learning. These results suggest that DMS and DLS neural networks gradually reorganize in different ways in order to progressively increase their control over the behavioral performance.

## Significance Statement

Our study helps understanding the role of the dorsomedial (DMS) and DLS during the acquisition and optimization of a behavioral strategy. It is generally believed that the DMS mediates action-outcome associations, whereas the DLS supports habit behavior, but it is still unclear how these processes emerges during learning. To analyze the dynamic changes of DMS and DLS network activity across learning stages, we used a mathematical analysis combining single neuron firing rate and connectivity between neurons to decode rat behavior in a goal-directed spatial task. We demonstrated that both DMS and DLS activity supports behavioral performance throughout all learning stages, thus challenging the hypothesis of a gradual shift from DMS to DLS activity.

## Introduction

The dorsal striatum integrates sensory, motivational, and motor information transmitted by cortical and thalamic neurons, thereby enhancing the capacity to select actions that result in favourable outcomes, such as rewards, while also minimizing engagement in undesirable actions. Its anatomical projections from the cortex support a functional dissociation along the medio-lateral axis. The dorsolateral striatum (DLS) receives cortical inputs from the sensorimotor and premotor cortices, and was shown to sustain inflexible motor routines, stimulus-response learning and more generally habit behaviour (Dickinson and Balleine, 1994; Dickinson et al., 1995; Balleine and Dickinson, 1998; Graybiel, 1998; Packard and Knowlton, 2002; Yin et al., 2004, 2005; Balleine and O’Doherty, 2010; Hilario et al., 2012). In contrast, the dorsomedial striatum (DMS) is mainly targeted by associative and prefrontal cortices and supports goal directed behaviour, controlled by flexible action-outcome contingencies (Reig and Silberberg, 2014; Hintiryan et al., 2016; Hunnicutt et al., 2016; Hooks et al., 2018). Accordingly, DMS and DLS display different task-related activity. The DLS was shown to develop preferential firing at the start and end of action sequences (“task bracketing”) (Jog et al., 1999; Barnes et al., 2005; Thorn et al., 2010; Smith and Graybiel, 2013) but see Sales-Carbonell et al. (2018), whereas the DMS mainly activate at cue onset and movement onset, as well as with changes in reward prediction (Stalnaker et al., 2012) or changes in the reward delivery contingency (Regier et al., 2015). Even though this functional dichotomy seems fairly well established throughout the literature, the gradual shift from the activity of the DMS in the early learning stages to the DLS in the automation phase has been questioned in the past few decades. Lesion and inactivation studies (Yin et al., 2004, 2005, 2009; Durieux et al., 2012) were the first to suggest that DMS gradually disengages in favour of the DLS during learning. This evidence is also supported by some electrophysiological studies (Thorn et al., 2010). In contrast, Vandaele et al. (2019) has recently shown that both striatal regions remain active long after the initial acquisition, when skilled performance is consolidated. One hypothesis that could explain such discrepancy is that DMS and DLS undergo region-specific network reorganization during learning, as suggested by Badreddine et al. (2022). This hypothesis could be tested by analyzing neural network properties in the DMS and DLS and their ability to decode behavior.

The goal of our study is to capture the dynamics of DMS and DLS neural activity across learning at the level of single cells and neural networks. To do so, we monitored DMS and DLS neural activity in a continuous spatial alternation task. In this task, the rats learn to continuously alternate between the left and right arms of a T maze using the spatial cues available in the room. No specific cue provides any behavioral instruction, thus limiting experimentally defined discrete actions that possibly influence “task bracketing” activity. Moreover, this task requires the activity of both DMS and DLS from the beginning of learning, as it has been shown in a previous study (Moussa et al., 2011). It is therefore particularly suited for the study of DMS/DLS interplay during learning phases. DMS and DLS single neuron activity was recorded from the first day rats were exposed to the task until they reached and maintained a stable performance. We first monitored task-related activity across the different learning phases (from early acquisition to stable performance). Then, we estimated DMS and DLS neural network functional properties using classical methods based on either firing rate or neural synchronization, but also combining the two measures using the Hawkes model. This model allows to reconstruct directed connectivity graphs of neurons, thus providing a functional connectivity network that was used to decode animal behavior. Overall, the results suggest that both DMS and DLS neural networks are engaged during all learning stages, but they undergo a differential gradual reorganization over time. Moreover, the results point to the importance of investigating neural network properties in addition to single neuron coding abilities, in order to better understand how brain structures participate to learning processes.

## Materials and Methods

### Animals and surgery

Seven male Long-Evans rats (Janvier, Le Genest-St-Isles, France) weighing 300–350 g were housed in individual cages (40 cm long × 26 cm wide × 16 cm high) with food and water ad libitum and maintained in a temperature-controlled room (20°C + / − 2). One week after their arrival, animals were handled daily by the experimenter for 7 days. 4 animals were then implanted with tetrodes aimed at either the left (3 rats) or the right (1 rat) DMS at the following coordinates: AP: ±1 mm, ML: ±2.2 mm from the midline, DV: −3 mm below the dura. Two animals were implanted with tetrodes aimed at the left DLS and one rat was implanted bilaterally at the following coordinates: AP: ±1 mm, ML: ±3.7 mm from the midline, DV: −3 mm below the dura (Extended Data Fig. 1-1). The surgery was performed under sterile conditions and under general anaesthesia (Ketamine 75 mg/kg (Imalgene 1000, Merial, France)/Medetomidine 0.25 mg/kg (Domitor, Janssen, France)). As postoperative treatment, the rats were injected with antibiotic (Clamoxyl, 150 mg/kg) and analgesic (Tolfedine, 4 mg/kg). After surgery, the rats were given 5–7 days of recovery. They were then subjected to a food deprivation program that kept them at 90% of their body weight during behavioral testing. All experiments were performed in accordance with the National Institute of Health’s Guide for Care and Use of Laboratory Animals (NIH Publication no. 80–23) revised in 1996 for the UK Animals (Scientific Procedures) Act of 1986 and associated guidelines or the Policy on Ethics approved by the Society for Neuroscience in November 1989 and amended in November 1993 and under veterinary and National Ethical Committee supervision (French Agriculture Ministry Authorization)

### Microdrives and recording setup

Four tetrodes formed a bundle threaded through a piece of stainless-steel tubing. Each tetrode consisted of four twisted 25 μm nichrome wires. The connector, tubing and wires could be moved downwards by turning the drive screw assemblies cemented to the skull. Cable was connected to the rat’s headstage, which contained a field effect transistor amplifier for each wire. The signals from each tetrode wire were amplified 10,000 times, bandpass filtered between 0.3 and 6 kHz with Neuralynx amplifiers (Neuralynx, Bozeman, MT, USA), digitised (32 kHz) and stored by the DataWave Sciworks acquisition system (DataWave Technologies, Longmont, CO, USA). A red light-emitting diode (LED) attached to the head assembly was used to determine the position of the rats. The LED was filmed by a CCD camera mounted on the ceiling above the maze, and its position was tracked at 25 Hz by a digital point tracker.

### T maze apparatus

**Figure 1.**
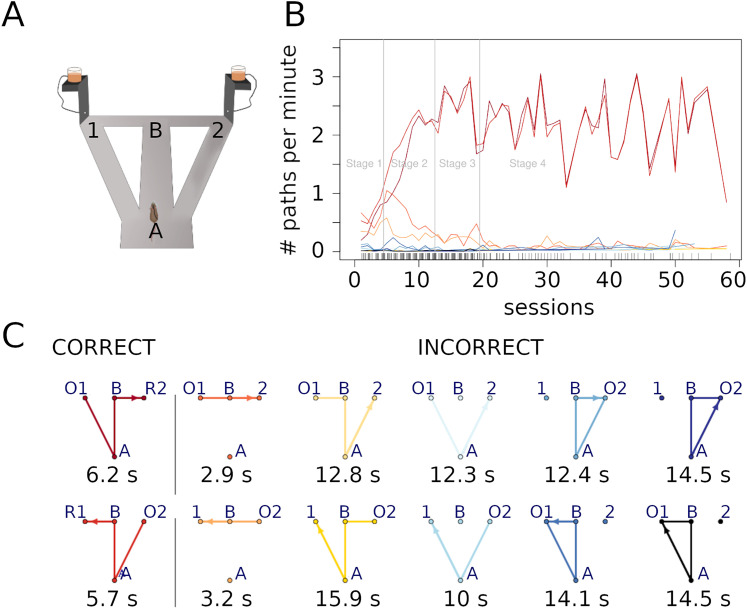
Behavioral data. ***A***, Sketch of the maze with the two intersections *A* and *B* and the two feeder’s locations 1 and 2. ***B***, Number of paths per minutes across learning stages. Each line corresponds to the average number of paths (correct and incorrect) taken by all rats across the different learning stages; path identities are indicated with the same colors as in ***C***. ***C***, Drawings of the 12 considered paths, including the 2 correct paths (red colors) in the first column and the 10 incorrect paths (orange to blue) in the second column. Each path begins with an Onset O (1 or 2 depending on the feeder location). Only the correct paths end with a Reward R (1 or 2 depending on the feeder location). Under each path, the corresponding average duration in seconds for all rats and all sessions is shown. Extended Data Figure 1-1 shows a reproduction of tetrode positions and recording sites in the DMS and DLS. Extended Data Figures 1-2 and 1-3 show the classification of neuron types, and the estimation of spike sorting quality, respectively.

The T-maze consisted of four 10 cm wide, grey-painted wooden tracks (with walls of 2 cm height on each side), a 100 cm long central stem, a 100 cm long crossbar forming the two goal arms, and two additional tracks each connecting the distal end of one goal arm to the base of the central stem. The reward wells were located at the distal end of each choice arm. Food rewards (45 mg sugar pellets) were released from two feeders (MedAssociates) mounted above the wells and activated by remote manual switches. The maze was elevated 40 cm off the ground on a metal frame. The apparatus was illuminated by four symmetrical spotlights (40 W) mounted on the ceiling. A centered radio above the maze was used to mask uncontrolled disturbing sounds and the experimenter was located in an adjacent room. Behavioral training was performed as follows. After one week of recovery period from surgery, the rats were familiarized with the maze in daily 20-minutes sessions for two days, during which they were allowed to freely explore the apparatus and collect randomly scattered sugar pellets. Training began on the third day with either one or two 20-min sessions per day. Animals were gently placed at the intersection point labelled “A” in Figure 1*A* of the “continuous T-maze” and let free to explore each arm as they wished. In order to obtain a 15 mg sugar pellet, they had to run along the central stem and alternately enter the left or right arm of choice. Rats were submitted to one training sessions per day. Tetrodes were lowered by either 25 μm or 50 μm after each training session or every two sessions, for a maximal length of 2 mm (between 1 and 2 mm, Extended Data Fig. 1-1).

### Data analysis—behavior

Animal position (x/y coordinates) over time was sampled at 25 Hz. A preprocessing of the data, consisting mainly in clearing rapid head movements and reflection artifacts of the camera, was first performed. We identified 12 possible elementary paths (i.e., 2 correct paths and 10 incorrect paths) that rats could travel along the maze (Fig. 1*C*). Note that some rare portions of the trajectories were not classified as elementary paths and were removed from the analysis. These mainly include animals jumping out of the maze or jumping in a noncontiguous spot in the maze. Animal performance was expressed as the number of paths (either correct or incorrect) per minute travelled by each rat during the training sessions. Based on rat performance, we identified four successive learning stages : Stage 1, with a similar number of correct and incorrect paths; Stage 2, showing a progressive increase of the number of correct paths and a progressive decrease of the incorrect ones; Stage 3, in which the number of correct and incorrect paths stabilize; and finally Stage 4, showing almost exclusively correct paths (Fig. 1*C*). The duration of each path type was also scored.

### Data analysis—neuronal activity

Neural data from the four DMS implanted rats were also used in another study investigating rat learning strategies using credit assignment models (James et al., 2023). All the results reported in the present study is original.

#### Spike Sorting

Spike sorting was performed manually using the graphical cluster-cutting software Offline Sorter (Plexon). Units selected for analysis had to be well-discriminated clusters with spiking activity clearly dissociated from background noise. Units that were lost or whose waveform changed too much before the session was completed, were excluded. Units having interspike intervals <2 ms were removed due to poor isolation, as were cells with a peak firing rate <1 Hz. The quality of spike clusters was estimated by calculating the L-ratio, that was originally described by Schmitzer-Torbert and Redish (2004) and applied to striatal data. The L-ratio is a measure of how close noise, or non-unit, waveforms are to a cluster’s center. A low value indicates that the cluster is well separated (i.e., few noise waveforms close to the unit’s cluster). To test whether this measure was significantly different from random, for each cell we calculated a cumulative distribution of L-ratio measures from clusters drawn randomly from all spikes (excluding the cluster analyzed) detected within the recording session (1000 iterations per cell). From this curve, we extracted the percentile value corresponding to the L-ratio measure of the cell. A total of 650 cell clusters was accepted (293 DMS and 357 DLS units). Since the tetrodes were lowered at the end of the recording session, neural activity in each session should be collected from different neurons. However, if two neurons recorded in two successive sessions showed similar characteristics in their firing patterns, waveforms and clusters, they were included only once.

#### MSN and FSI classification

For each neuron we computed the log of the global firing rate, the peak-valley distance (PV) (i.e., the distance between the peak and next minimal value) and the peak width at half-height (W) from the digitalized waveforms. These three variables were next centered and renormalized. Then, we used the hierarchical clustering method with “Ward 2” distance to build the dendrogram, showing clearly the existence of 2 clusters (Extended Data Fig. 1-2). The k-means cluster algorithm with *k* = 2 was used to identify the clusters. The cluster with small firing rate and large PV and W corresponded to putative medium-spiny neurons (MSN), whereas points with large firing rate and small PV and W were allocated to the putative fast-spiking interneurons (FSI) cluster.

#### Identification of coding neurons

To identify coding neurons we performed three different analysis that were based on either temporal or spatial firing activity. In the first analysis, a task-event coding neuron was defined as a neuron showing significantly different firing activity during any of the six task-related temporal events (described below). For the second analysis (left/right coding neurons), we focused on the neural firing within the central stem of the maze with respect to whether the rat will subsequently chose the left- or right-goal arm. In this case, a coding neuron should activate differently for left and right turns. The third analysis allowed us to categorize path coding neurons as neurons showing significantly different firing activity between any two of the 12 paths described in the behavioral section. Path coding neurons were then used in the decoding analysis. The results from these analysis were used to compare the proportion of coding neurons across learning stages, brain areas (DMS–DLS) and types of neurons (FSI–MSN). Whatever the type of coding, the detection of coding neuron is the same and is described hereafter.

#### Chi-squared method for detection of coding neurons

The purpose of this statistical analysis is to test whether the firing activity is constant over all possible conditions (null hypothesis) or if it is significantly different in at least one given condition (i.e., a 100 ms bin or a particular path) with respect to all other possible conditions. These possible conditions will include the 30 temporal bins defining the six task events in the first analysis (i.e., five 100 ms-bins per event), or the two left and right paths for the second analysis, or the paths taken in a session for the third analysis. Given *N*_*k*_ the number of spikes produced in a given condition *k* = *k*, …, *K*, we compute the *p*-value of the chi-square test that decides if (*N*_1_, …, *N*_*K*_) is a multinomial distribution of parameters

$$p=\left(\frac{d_{1}}{d_{1}+\cdots +d_{K}},\ldots ,\frac{d_{K}}{d_{1}+\cdots +d_{K}}\right)$$

and

$$n=N_{1}+\cdots +N_{K},$$

with *d*_*k*_, the duration of the condition *k* in the session. The test gives a valid *p*-value if *np*_*k*_ ≥ 5 for all coordinates *k*. Therefore if it is not the case, we can either drop a condition or group together conditions. If despite that, then there is still not enough spikes, the corresponding neuron was removed from the analysis. All neurons with a Benjamini-Hochberg (BH) adjusted *p*-value lower than 0.05 were declared as coding for a particular set of conditions. To assess the difference in the proportion of coding neurons (see Table 2), we then used a generalized linear model (Bernoulli with probit link) to explain the coding character as a function of brain region, neuron type and learning stages (with cross effects Brain region-Learning stage and Brain region-neuron type). Each time, DLS, MSN and Stage 1 were used as references. In a preliminary study, firing rates and all other possible cross-effects were added to the model, but since no significant results even without correction were found, they have been discarded. The fact that the firing rate does not impact the coding character is in adequation with the chi-square detection method: as soon as there are enough spikes in total in the session to use the method, there is no bias *per se* toward largest firing rates, once the FSI character has been taken into account.

**Figure 2.**
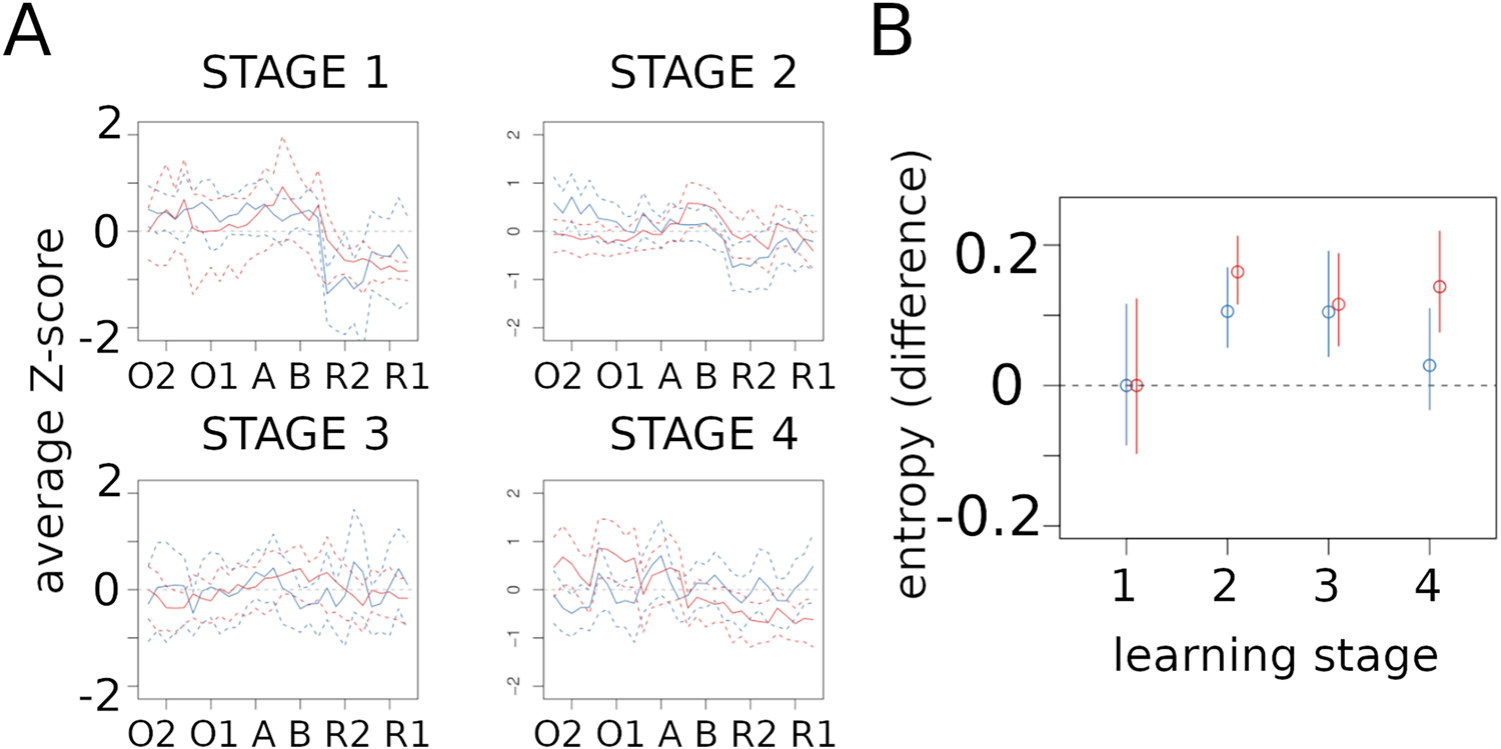
Z-score and Entropy. ***A***, Average z-score of “Task-event” coding neurons in DMS (red) and DLS (blue) in the four learning stages. The dashed lines represent the confidence intervals at level 0.05, corrected for multiplicity over the 8 curves and the 30 time intervals (Bonferroni). ***B***, Difference in Z-score entropy between learning stages for DMS (red) and DLS (blue) neurons. Stage 1 is used as reference. The vertical lines represent the confidence intervals at level 0.05, corrected for multiplicity over the 4 stages and the 2 brain regions. Extended Data Figure 2-1 shows how task-events were precisely defined.

- *Task-event coding neurons.* To identify event-based coding properties, we analyzed neural firing during specific time windows in four areas of the maze : the two intersections (depicted as A and B in Fig. 1*A*) and the two reward areas (depicted as R1 and R2 in Fig. 1*A*). We defined six 500 ms time events based on the passages through the different areas (see Extended Data Fig. 1-4*A*). For the two intersections A and B, we extracted the central 500 ms time window of the trajectory within the two areas. For the reward areas, we defined two different events: the reward obtention (R1 and R2 for the left- and right-goal arm) including the first 500 ms after the rat entered the reward areas, and the movement onset (O1 and O2 for the left- and right-goal arm) including the last 500 ms before the rat left the reward areas (Extended Data Fig. 1-4*B*). For each neuron we calculated the average firing rate during the 500 ms events by refining the time window in five bins of 100 ms. A task-event coding neuron is a neuron that has been detected by the chi-square method to show a firing rate activity in one 100 ms bin that is different over all 30 time bins (i.e., five bins for each of the six events). If the condition *np* ≥ 5 is not fulfilled, then we have grouped the 100 ms bins into six conditions, corresponding to six time windows of 500 ms around each event (intersections, rewards, onsets). If the condition is still unfulfilled, then the neuron is discarded.
  The z-score of an event coding neuron *n* in a 100 ms bin of time *t* is given by
  $$z_{n,t}=\frac{\;f_{n,t}-{\bar{\;f}}_{n,.}}{\sigma (f_{n,.})},$$
  where *f*_*n*,*t*_ is the firing rate of neuron *n* in bin *t*, ${\bar{\;f}}_{n,.}$ is the average over the bins and *σ*(*f*_*n*,._) the corresponding standard deviation over the bins.
  Then, we averaged the normalized (z-score) activity of DMS and DLS event coding neurons over each neuronal population–brain region*stage—(see Fig. 2*A*).
  We also estimated the entropy of the spike probability distribution in each population from the ensemble z-scores across learning stages by counting the number **Nz**_*I*_ of *z*_*n*,*t*_ in a given population that appears in an interval *I* of z-score given by segmenting [ − 3.5, 5.5] in bins of 0.5 length, then the entropy of the distribution of z-score was estimated by
  $$\sum_{I\;s.t.{Nz}_{I}\neq 0}\frac{{Nz}_{I}}{{Nz}_{tot}}\log\;\left(\frac{{Nz}_{I}}{0.5{Nz}_{tot}}\right),$$
  where **Nz**_*tot*_ is the total number of z-score in the population. Note that $\frac{{Nz}_{I}}{0.5{Nz}_{tot}}$ estimates the density of the z-score distribution inside interval *I*.
- *Neurons coding for left and right turns.* Here, we focused on DMS and DLS neural firing activity in the central stem of the maze (between A and B intersections, Fig. 1*A*) with respect to whether the rat will subsequently choose the left- or right-goal arm. Here, the chi-square method is performed only with two conditions and there is no grouping or dropping of conditions if *np* ≥ 5 is not fulfilled. In this case, the neuron is just discarded.
- *Neurons coding for paths.* Firing activity in DMS and DLS during any of the 12 possible paths was calculated. We use again the chi-square method to identify significant spike activity in one of the 12 paths over all paths. If any of the path *k* does not satisfy *np*_*k*_ ≥ 5, this condition is simply left out. This is in particular the case if *d*_*k*_ = 0, meaning that this path is not taken by the rat during the session. If even with only two paths left, the condition *np* ≥ 5 is not satisfied, the neuron is discarded.

#### Neuronal synchronization at maze intersections

For each learning session, we performed a trial shuffling/permutation analysis to detect synchronization of spiking activity between pairs of simultaneously recorded neurons at the intersection areas of the maze A and B (see Albert et al., 2015, 2016). More precisely, for any pair of neurons simultaneously recorded, we isolated the number of times the rat passed through A or B and for each passage we extracted the central 500 ms window, defined as a trial. The number of coincident spiking activity observed within 20 ms in each trial was compared to the empirical distribution obtained by computing 50 000 permutations of the trials, to obtain a *p*-value. We then calculated the cumulative distribution functions (c.d.f.) of all *p*-values from pairs of neurons in the DMS and the DLS across learning stages. We quantified the deviation of the c.d.f. from the diagonal by a Kolmogorov–Smirnov test of uniformity and the comparison between DMS and DLS c.d.f.s by a two-sample Kolmogorov–Smirnov test.

#### Decoding behavior using Poisson and Hawkes models

Decoding of rat behavior (i.e., choosing a particular path among the 12 identified, Fig. 1*C*) was implemented using Poisson and Hawkes models. We chose Poisson model since neural firing is generally modeled in that way. In addition, we used the less conventional multivariate Hawkes model because it includes not only firing rate but also the strength and the directionality of the interaction between pairs of neurons (Lambert et al., 2018; Reynaud-Bouret et al., 2021). We included in the analysis exclusively DMS and DLS “Full Paths” neurons. This allows to obtain a sufficient number of spikes for each path to apply the Hawkes method. In addition, this prevents the method to be diluted by a different proportion of noncoding neurons in each area. The same analysis was also applied to “Left-Right Turns” neurons, and to FSI and MSN neurons, separately. All sessions with less than 2 “Full path” coding neurons were excluded. In addition, the rat has to perform at least two different paths (among the 10 paths described in Fig. 1), and each path needs to be repeated three times. In total, we analyzed 171 sessions (among 206).

The Poisson model is defined by an intensity (instantaneous firing rate) of neuron *n* in condition (path) *k* given by

$$\lambda_{n,k}(t)=\nu_{n,k}.$$

The parameter *ν*_*n*,*k*_ represents the firing rate of neuron *n* in condition *k*. Hence if *N* is the total number of path coding neurons in the session, the Poisson model has *N* parameters per condition.

The Hawkes model is defined by an intensity of neuron *n* in condition (path) *k* given by

$$\lambda_{n,k}(t)=\mu_{n,k}+\sum_{m=1}^{N}\sum_{T\;\mathrm{spikes}\;\mathrm{of}\;\mathrm{neuron}\;m,T<t}h_{m\to n}(t-T).$$

The parameter μ_*n*,*k*_ represents the spontaneous firing rate of neuron *n* in condition *k*. In addition, the interaction *h*_*m*→*n*_ quantifies the impact of one spike of neuron *m* at time *T* on the apparition of a spike on neuron *n*. The functions *h*_*m*→*n*_ are decomposed into 6 plateaux of length 10 ms each associated with a different value *a*_*m*,*n*,1_, …, *a*_*m*,*n*,6_, so that the value of *h*_*m*→*n*_ between 10 and 20 ms is for instance *a*_*m*,*n*,2_. The strength of an interaction is then measured by $|h_{m\to n}|=\sum_{i=1}^{6}|a_{m,n,i}|.$ In total, *N* + 6*N*^2^ parameters per condition are necessary for a Hawkes process. In particular, it includes self-interactions.

In each model, the parameters are estimated by minimizing the least-square contrast that is

$$C(\lambda_{n,k})=-2\sum_{T\;\mathrm{spikes}\;\mathrm{of}\;\mathrm{neuron}\;n}\lambda_{n,k}(T)+\int \lambda_{n,k}{\left(t\right)}^{2}dt.$$

More precisely, for Figure 4, the sum and integral above are considered over all time *T* or *t* such that condition *k* holds. The estimated parameters *ν*_*n*,*k*_ for the Poisson and μ_*n*,*k*_ for the Hawkes are given by the color of the nodes. The strength of the interaction *h*_*m*→*n*_ is given by the color of the edge *m* → *n*.

To test for the presence or absence of an edge in the Hawkes estimated networks we used a factorial analysis. First we assigned a value of 1 or 0 to an edge if the strength (*L*^1^ norm) of the interaction is greater or lower than 0.17, corresponding to the 70% quantile of all the strengths that have been estimated. A generalized linear model (Bernoulli with probit link) was then applied on the resulting Bernoulli variable (one per edge) with factors : target (MSN/ FSI), source (MSN/FSI), learning stage, region (DLS/DMS). We included all double cross effects, and the triple cross effect source*target*region and source*target*stage. The reference is target MSN, source MSN, stage 1 and DLS. All *p*-values were corrected for multiplicity by Benjamini Hochberg.

The computation of the decoding power is more intricate. In each session, we consider only the paths that are taken at least 3 times. We defined as a trial each single path (and the corresponding neural activity) taken by the rat. So for condition (path) we have at least 3 trials. For each condition *k*, we computed estimators of the two models by using only 2/3 of the trials, leading to estimators of the intensity ${\hat{\lambda }}_{n,k}$. To do so, it is sufficient to restrict the integral and summation in the least-square contrast *C*(*λ*_*n*,*k*_) = *C*_2/3_(*λ*_*n*,*k*_) to time inside 2/3 of the trials. For each condition *k*, it remains 1/3 of the trials. For each remaining trial, we compute the least-square contrast $C({\hat{\lambda }}_{n,k^{\prime }})=C_{trial}({\hat{\lambda }}_{n,k^{\prime }})$, for all condition *k*′, where now the time in the sum and integrals in *C* is restricted to this precise trial. Therefore, $C_{trial}({\hat{\lambda }}_{n,k^{\prime }})$ measures the similarity between the model estimated in the condition *k*′ and the spike train of the trial under inspection, which is in condition *k*. The estimated condition of a given trial is therefore given by

$$\hat{k}=\arg\;\min_{k^{\prime }}C_{trial}({\hat{\lambda }}_{n,k^{\prime }}).$$

The decoding power of a model is the proportion of good guess, that is the proportion of trials for which $\hat{k}=k$.

The decoding power strongly depends on the number of paths (Nb Paths) within a session. Indeed, if there is not enough information in one path to decode rat behavior, the decoding power should be equivalent to 1/Nb Paths. Therefore, we used this value as reference (i.e., random guess), that was used to assess the differences in decoding power between Poisson and Hawkes model in Figure 5.

Then , we calculated the decoding power for each striatal region across the four learning stages. As previously noted, the decoding power depends on the number of paths (Nb Paths) but also on the number of (path coding) neurons (Nb Neurons) and their average firing rates (Firing). Moreover, the encoding power is a quantity in [0, 1] that is best modeled by a beta variable. Therefore, we performed a beta regression with probit link to test the effect of Nb Paths, Nb Neurons, Firing, Striatal Region (i.e., DMS/DLS), Learning Stage and cross effects Region/Stage, to disentangle the effects of interest (Region, Stage) and the other auxiliary effects that we need to take into account.

## Results

### Behavioral performance

Animal performance during each session was expressed as the number of paths traveled per minute. Paths consisted of 2 correct and 10 incorrect trajectories (see Materials and Methods Section and Fig. 1*C*,*D*) and were defined based on the previous study (James et al., 2023). Performance evolution across training sessions was similar in all rats, with a gradual increase of the number of correct trajectories and a decrease of the number of incorrect trajectories (Fig. 1*B*). Based on the average rate of correct and incorrect paths (i.e., number of paths traveled per minute), we identified four successive stages of learning: stage 1, with the similar rate of correct and incorrect paths; stage 2, showing a progressive increase of the rate of correct paths and a progressive decrease of the rate of incorrect paths; stage 3, in which the rate of correct and incorrect paths stabilizes; and finally stage 4, showing almost exclusively correct paths.

10.1523/ENEURO.0436-23.2024.f1-1 Figure 1-1 Reproduction of tetrode positions and recording sites in the DMS and DLS. A: Photos of Nissl-stained coronal sections from two representative rats implanted in the DLS (left panel) and DMS (right panel). The tetrode tracks are visible in both sections. The vertical black bars represent the lengths of striatal tissu from which neural recordings were performed. B: Representation of the recording sites from all rats (DLS in blue and DMS in red). Download Figure1-1, TIF file.

10.1523/ENEURO.0436-23.2024.f1-2 Figure 1-2 FSI-MSN classification. A: Dendogram of the hierarchical clustering (method “Ward D2”) of neurons based on waveform (wf) properties (Peak-Valley distance (PV), Width at mid-height (W)) and firing rate. Two clusters are identified using K-means method (with *K *= 2). B: 3D representation of wf properties and firing rate of the two clusters. Fast Spiking Interneurons (FSI) and Medium Spiny Neurons (MSN) are represented in orange and green, respectively. A total of 451 MSN and 199 FSIs have been identified. No significant difference in the proportion of FSI/MSN was observed between DMS and DLS (p-value = 0.97). Download Figure 1-2, TIF file.

**Figure 3.**
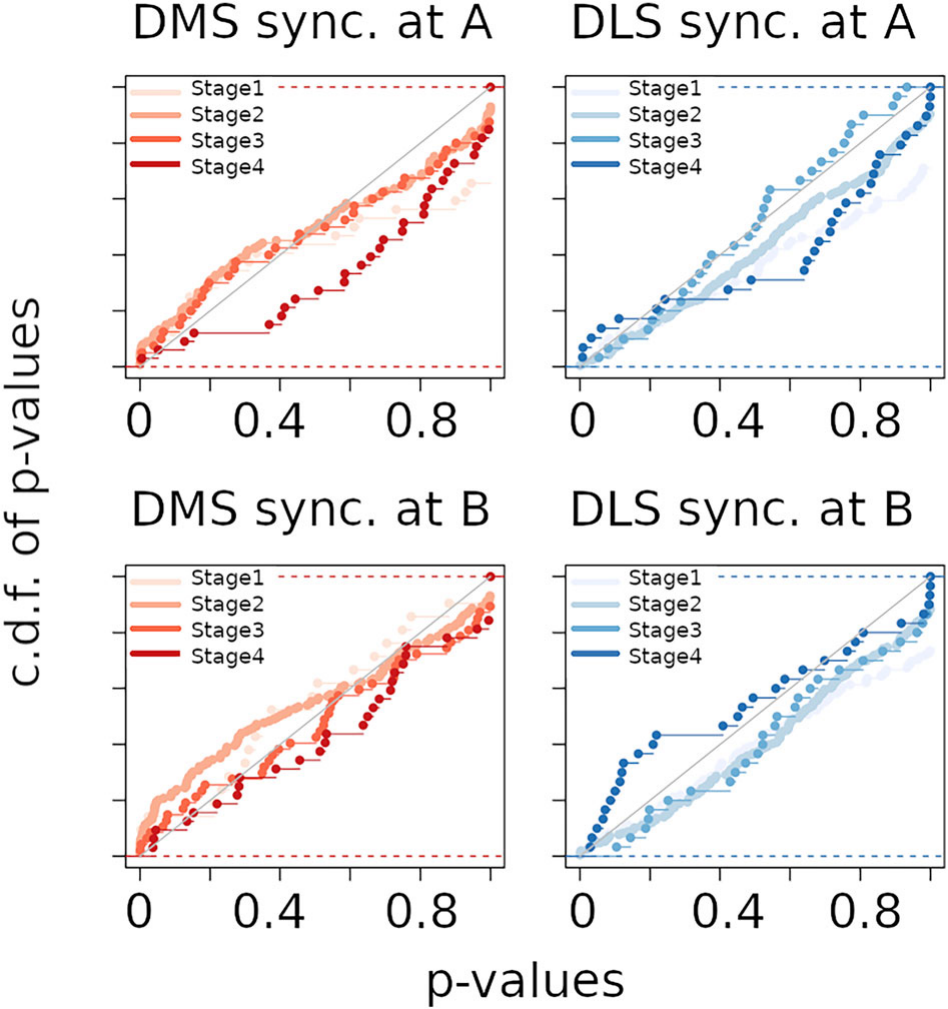
Synchronization analysis at maze intersections *A* and *B*. Synchronization between pairs of neurons was detected in 500 ms time windows centered on the two maze intersections (*A* and *B*). Coincidences count was statistically compared to the empirical distribution of 50,000 trial permutations. Cumulative distribution functions (c.d.f.) of the obtained *p*-values have been plotted as a function of the brain region (red for DMS and blue for DLS), intersection A or B and learning stages. A one sided Kolmogorov–Smirnov test of uniformity have been performed to compare all c.d.f. curves (16 in total). In addition, for each learning stage and maze intersection a two-sample one sided Kolmogorov–Smirnov test has been performed. All *p*-values were adjusted for multiplicity by Benjamini-Hochberg method. Note that c.d.f. of DMS *p*-values in stage 2 at intersection *B* is significantly above the diagonal (adj. *p*-val. = 8.6 10^−3^). Moreover, in stage 2, c.d.f. of DMS *p*-values at A and B are significantly above c.d.f. of DLS *p*-values (intersection A: adj. *p*-val. = 0.027; intersection B: adj. *p*-val. = 1.7 e–04).

10.1523/ENEURO.0436-23.2024.f1-3 Figure 1-3 Estimation of spike sorting quality for FSIs and MSNs. From left to right: distributions of the percentile values of L-ratio measures (with k-means density curves in black) for all FSIs (left) and MSNs (right) recorded from DLS (in blue) and DMS (in red); examples of spike clusters and spike autocorrelograms from 2 neurons for each category (FSI - DLS and DMS, MSN - DLS and DMS). Autocorrelograms were constructed using 20  ms windows and 0.5  ms bins. Download Figure 1-3, TIF file.

### Task-event coding neurons

We recorded 293 neurons in 4 DMS-implanted animals and 357 neurons in 3 DLS-implanted animals over a total of 206 training sessions (Extended Data Fig. 1-1). On the basis of the firing rate and the waveform properties (i.e., peak-valley distance and peak width at mid-height of the peak), cluster analysis revealed the presence of two clear clusters (Berke et al., 2004) (see Materials and Methods section and Extended Data Fig. 1-2 and 1-3). Neurons with low firing rates and large waveforms were classified as putative medium spiny neurons (MSN, GABA-ergic projection neurons), whereas the other group was identified as putative fast-spiking interneurons (FSI, GABA-ergic interneurons). A total of 451 MSNs and 199 FSIs were found. A chi-square test of independence showed no significant difference in the proportion of FSI/MSNs between DMS and DLS (*p*-val. = 0.97).

To analyze possible differences in firing activity in DMS and DLS neurons across learning stages, we first used a classical analytical approach and categorized neurons as coding task events based on their firing activity in specific time windows. We identified six 500 ms events: the two onsets O1 and O2 corresponding to the beginning of each path, the two intersections A and B of the maze and the two rewards R1 and R2, corresponding to the end of the correct paths (just before receiving the reward) (see Extended Data Fig. 2-1 for a precise definition). Each of the six 500 ms events was divided into five 100 ms intervals. We classified as task event coding each neuron whose firing activity in one 100 ms interval varied significantly from all other intervals using a chi-squared detection method (see Methods Section for a description of the statistical identification procedure) (Table 1). 

**Table 1.** Neurons coding for task events (“Task-event”), neurons coding for left and right turns (“Left-Right Turns”) and neurons coding for the entire paths (“Full Paths”)

Coding type	Task-event	Left-Right Turns	Full Paths
$\frac{\#\text{Coding}}{\#\text{Noncoding}}\;\mathrm{DMS}|\mathrm{DLS}$	$\frac{192}{47}|\frac{136}{87}$	$\frac{102}{144}|\frac{42}{172}$	$\frac{226}{61}|\frac{182}{161}$
DMS Coeff. (adj. *p*-val.)	1.27 (0.06)	1.12 (0.05)	0.86 (0.03)
FSI Coeff. (adj. *p*-val.)	−0.52 (0.03)	–	–
Stage 2 Coeff. (adj. *p*-val.)	–	–	–
Stage 3 Coeff. (adj. *p*-val.)	–	0.97 (0.06)	–
Stage 4 Coeff. (adj. *p*-val.)	0.68 (0.07)	0.98 (0.06)	–
Cross-Effect DMS:FSI Coeff. (adj. *p*-val.)	0.57 (0.08)	–	–
Cross-Effect DMS:Stage 2 Coeff. (adj. *p*-val.)	–	–	–
Cross-Effect DMS:Stage 3 Coeff. (adj. *p*-val.)	–	−1.06 (0.08)	–
Cross-Effect DMS:Stage 4 Coeff. (adj. *p*-val.)	–1.86 (0.03)	–	−0.82 (0.08)

First Line: On the top, number of coding neurons and below, number of noncoding neurons, in the DMS (left) and DLS (right). The remaining lines report the results of a generalized linear model (see method section). The reference corresponds to DLS, MSN and learning stage 1. All *p*-values have been adjusted for multiplicity by Benjamini-Hochberg method. Only adjusted *p*-values <0.1 corresponding to a False Discovery Rate less than 10% have been reported with their associated coefficient.

We found in total (i.e., all learning stages and neuron types–FSI and MSN–included) 65,5% of “Task-event” neurons in DMS (16% noncoding neurons) and 38% in DLS (24% noncoding). 18.5% of DMS and 38% of DLS could not be classified because of too low firing rate. The Pearson’s Chi-squared test showed that the proportion of coding neurons is significantly higher in the DMS compared to the DLS (*p*-val. = 7.57e–06). The results from the general linear model (with references: DLS and MSN) confirmed this effect (see the first column of Table 2). The proportion of “Task-event” neurons in the DMS is greater than in the DLS (adj. *p*-val. = 0.06). Interestingly, this proportion is stable across learning stages except for the stage 4 during which the proportion of DLS “Task-event” neurons increases (adj. *p*-val. = 0.07 with positive coefficient (coeff. = 0.68) at learning stage 4), while the proportion of DMS “Task-event” neurons decreases (cross-effect adj. *p*-val. = 0.03, with negative coefficient (coeff = 0.57 − 1.86 = −1.29)). Finally, in the DLS, MSNs tend to code for task events more than FSIs (adj. *p*-val. = 0.03 with a negative coefficient (coeff. = −0.52) ), whereas in the DMS the difference between FSIs and MSNs is marginal (cross-effect adj. *p*-val. = 0.08 with coeff. = −0.52 + 0.57 =  0.05).

**Table 2.** Statistical analysis of the strength of the connections in DMS and DLS reconstructed networks

Factors	Coefficient	Adjusted *p*-values
target FSI	1.16	<10^−7^
source FSI	−0.83	1.5 × 10^−4^
Stage 2	0.15	0.045
Stage 3	0.43	1.8 × 10^−4^
Stage 4	0.39	6.3 × 10^−4^
DMS	0.27	1.8 × 10^−4^
Cross Effect target FSI: Stage 4	−1.04	1.5 × 10^−4^
Cross Effect target FSI: DMS	−0.27	0.033
Cross Effect source FSI: DMS	0.42	0.033
Cross Effect Stage 2: DMS	−0.19	0.045
Cross Effect Stage 3: DMS	−0.66	3.2 × 10^−6^
Cross Effect Stage 4: DMS	−0.54	1.8 × 10^−4^

The reference is : target MSN, source MSN, stage 1 and DLS. All *p*-values were corrected for multiplicity by Benjamini Hochberg. Only the adjusted *p*-values smaller than 0.05 are reported in the table.

Figure 2*A* shows the normalized average firing activity and the corresponding confidence intervals at the different task events, for neurons in the DMS (red) and DLS (blue) during the four learning stages. At the beginning of learning, during stage 1, the activity of DMS and DLS neurons decreases similarly during the R1 and R2 events. In stage 2, as the behavioral performance improves, DMS and DLS neural activity starts to diverge. DMS neurons increase their firing rate particularly at the intersection B of the maze, before the rat turns into the left or right arm, whereas no significant change was observed at the intersection A, yet implying similar turning behavior. This result possibly indicates an involvement of the DMS in the implementation of the learning rule. As for the DLS, neurons change firing rate specifically at the reward areas during both R and O events. These modifications are similar to the DLS “task bracketing” activity reported in previous studies (Thorn et al., 2010; Cunningham et al., 2021). Finally, no specific task-related activity is observed in the DLS during the later stages of learning, whereas the DMS tends to show some activity at action boundaries similar to DLS, during stage 4, as also shown by Vandaele et al. (2019).

10.1523/ENEURO.0436-23.2024.f2-1 Figure 2-1 Timeline of a typical training session. **A**: The maze has been divided in boxes. The black line represents the trajectory of the animal during one example session. The feeders are located in the boxes 1 and 2. Maze intersections are located in boxes A and B. **B**: Each 500  ms task event (centered on the onsets, intersections and reward locations) was divided in five 100  ms bins. This leads to a total of 6 * 5 = 30 time bins. Download Figure 2-1, TIF file.

To test whether these changes in neural activity during task events may reflect changes in coding capacity across learning stages, we estimated the z-score entropy from the firing rates of the “Task-event” neurons in the DMS and DLS. A decrease in entropy reflects a decrease in the randomness of the (re-normalized) firing rate in the neural population, which is classically interpreted as an increase in the coding ability of the neuronal population (Thorn et al., 2010). As shown in Figure 2*B*, the entropy of both DMS and DLS firing activity increases between stages 1 and 3, which may suggest a decrease in coding capacity during task acquisition for both areas. However, in contrast to the DMS, the entropy of the DLS tends to decrease in stage 4 to a level similar to that observed in stage 1. This may indicate that the DLS, but not the DMS, is involved in late learning stages following overtraining, as previously suggested (Tang et al., 2009). However, given the overlapping confidence intervals, it is difficult to draw a clear conclusion. It should also be noted that in this task animals are free to perform any trajectory and no specific instructions are given at any time. Thus, an event-based analysis may not be the most appropriate to reveal learning-related changes in neural activities. We therefore analyzed DMS and DLS capacity to code for specific paths (rather than events) across learning stages, as presented in the next paragraph.

### Neurons coding for left and right turns

In classical event-based analysis, a change in firing rate is considered as a code for a specific event in the task (i.e., movement onset, reward, choice), but not for the task itself (here for example dissociating left and right turns). For this reason, we compared the firing activity of each neuron in the central stem (from A to B intersections) between left-turn and right-turn trials, including correct and incorrect paths. If the firing activity is significantly different between left-turn and right-turn trials, the neuron is classified as a left/right turns coding neuron (see the Methods section for a full description of the statistical method). Table 1 (second column) summarizes the results of the statistical analysis. As for the “Task-event” neurons, we detected significantly more “Left-Right Turns” neurons in the DMS than in the DLS (adj. *p*-val. = 0.05), but no difference between FSIs and MSNs. Moreover the proportion of coding neurons tends to increase during learning stages 3 and 4 in the DLS (coeff. 0.97 or 0.98 and adj. *p*-val = 0.06), whereas it is less clear for the DMS (stage 3: cross effect adj. *p*-val. = 0.08, coeff = 0.97 − 1.06 = −0.09).

We then focused on neural synchronization as a marker of task encoding (Grammont and Riehle, 2003; Avital et al., 2013) and we calculated for each pair of DMS or DLS neurons, recorded simultaneously in each session, the probability of being synchronized at intersections A and B. We analyzed a total of 1821 pairs of neurons. The cumulative distribution functions (c.d.f.) of all *p*-values (one per neuron pair) are plotted in Figure 3 for DMS (red) and DLS (blue) neurons, at each learning stage. If the neuron pairs are independent, then the c.d.f. should be below the diagonal; any positive deviation from the diagonal, especially for small *p*-values, reflects a lack of independence (i.e., the neurons are synchronized). The Kolmogorov–Smirnov test revealed a significant deviation from the diagonal for DMS neurons at stage 2, particularly at intersection B (adj. *p*-val. = 8.6 e–03), whereas no significant effect was observed for DLS neurons at all stages. We then compared the c.d.f. of each learning stage between DMS and DLS to assess whether DMS neurons were more synchronized than DLS neurons. We found that in stage 2, the c.d.f. for DMS neurons was significantly higher than the c.d.f. for DLS neurons at both intersections A (adj. *p*-val. = 0.027) and B (adj. *p*-val. = 1.71e–04). These results indicate that, overall, DMS neurons tend to fire simultaneously more than DLS neurons during initial learning. In addition, DMS neurons are significantly synchronized at intersection B of the maze, before the rat turns into the left- or right-goal arm, especially during initial learning.

Overall, these results demonstrate that DMS neurons code for left and right turns, particularly during initial learning, an activity that may support the acquisition of the task rule. This hypothesis is reinforced by the observation that DMS neurons are synchronized during initial learning, particularly before the rat chooses the left- or right-goal arm. In contrast, no specific synchronization pattern was observed in the DLS at any learning phase.

### Functional connectivity and decoding power of DMS and DLS networks

We then asked whether the previously identified coding properties were decisive for decoding animal behavior. In other words, we asked whether the information provided by neural activity was sufficient to guess the path taken by the rat (among the 12 identified). Neural activity is traditionally modeled by a Poisson process based on the firing rate. However, we have shown that the synchronization of neurons, in addition to the firing rate, also reflects their coding capacity: we therefore need a model that takes into account both single neuron firing activity and neural interactions. We chose to use the multivariate Hawkes model, which includes neural firing rate, as well as the strength and direction of the interactions between neurons (Lambert et al., 2018; Reynaud-Bouret et al., 2021). We focused our analysis on the neurons showing significantly different firing activity for at least one path (“Full Paths” neurons : 181 DLS and 226 DMS neurons, 171 sessions) (see third column of Table 1). This choice was motivated by the fact that our ultimate goal is to use the reconstructed functional networks to decode which path among the 12 paths is traveled by the animal. It is therefore logical to focus on this category of coding neurons. Moreover, “Full Paths” is the category including the highest number of coding neurons, thus allowing to analyse relatively large neural networks.

**Figure 4.**
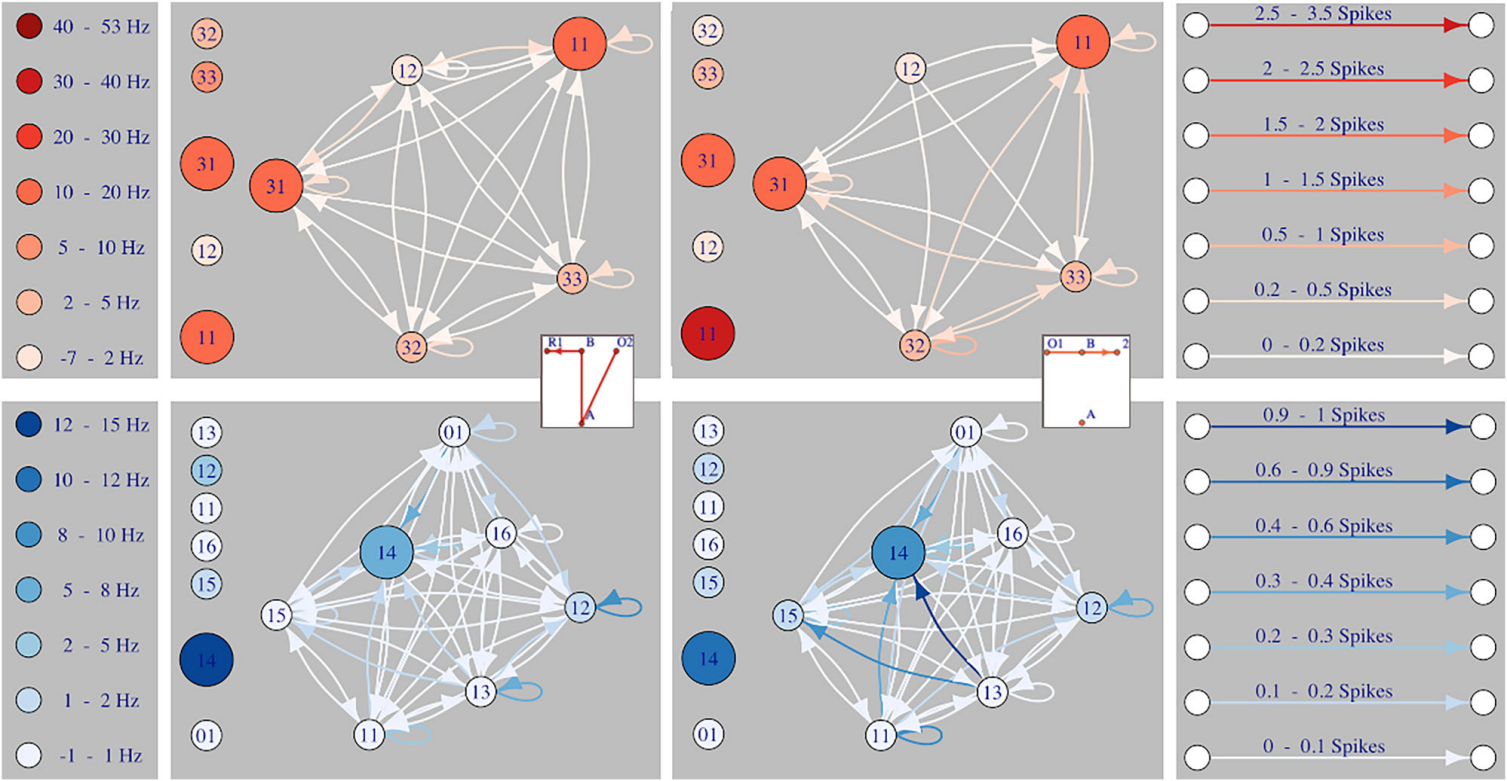
Examples of estimated network models of “Full path” neurons recorded from one DMS-implanted rat (red) and one DLS-implanted rat (blue) during one training session (session 6 for both). Left panel: color codes for the average firing rate. Middle panels: 4 network models (Poisson model on the left, Hawkes model on the right) of the same neurons during the execution of two different paths (a correct path on the left and a straight path on the right) from the same session. Each node represents one neuron; the size of the node represents the neuron type (big circles for FSIs and small circles for MSNs); the color of the node represents either the average firing rate for the Poisson model or the spontaneous part for Hawkes model (i.e., the baseline firing activity with all interaction functions canceled); the color and direction of the lines represent the interaction function. Right panel: color codes for the interaction function, with the number of spikes modified by this interaction. Extended Data Figures 4-1 and 4-2 show more examples of estimated network models from DLS and DMS, respectively.

Figure 4 shows the interaction graphs reconstructed from the Hawkes model versus the Poisson model (vertical line) during a representative session for a DMS–(red) and a DLS-implanted rat (blue) (more examples of interaction graphs from different DMS- and DLS-implanted rats and different learning sessions are provided in Extended Data Figs. 4-1 and 4-2). The reconstructed graphs for two different paths from the same session are shown in the same line. Neuron identity (FSI or MSN) is represented by the size of the nodes (small circles for MSNs and large circles for FSIs). The color code represents either the firing rate for the Poisson model or the spontaneous part for the Hawkes model (i.e., the baseline activity with all interaction functions canceled). For the Hawkes model, the direction and strength of the interactions between two neurons are represented by the direction and color of the arrows, respectively. In this example, we can clearly notice that, for the same set of neurons, the graphs for the two paths are different while their firing activity is roughly the same.

10.1523/ENEURO.0436-23.2024.f4-1 Figure 4-1 Examples of estimated network models for DLS ‘Full path’ neurons from two different rats and two different learning sessions. Upper panel: session 5 - learning stage 2. Lower panel: session 36 - learning stage 4. From left to right: color codes for the average firing rate ; 4 network models of the same neurons reconstructed for 4 different paths (Poisson model on the left, Hawkes model on the right); color codes for the interaction function. Download Figure 4-1, TIF file.

10.1523/ENEURO.0436-23.2024.f4-2 Figure 4-2 Examples of estimated network models for DMS ‘ Full path’ neurons from three different rats and three different learning sessions. Upper panel: session 2 - learning stage 1. Middle panel: session 19 - learning stage 3. Lower panel: session 30 - learning stage 4. From left to right: color codes for the average firing rate ; 4 network models of the same neurons reconstructed for 4 different paths (Poisson model on the left, Hawkes model on the right); color codes for the interaction function. Download Figure 4-2, TIF file.

We performed a statistical analysis to test which factors could influence the strength of the interactions between the nodes. We first assigned a value of 1 or 0 to the strength values if they were greater or smaller than the 70% quantile of all estimated strengths, respectively. Then, we used a generalized linear model to estimate the influence of the neuron identity (FSI or MSN, both as source or target of the strong interactions), the learning stage (from 1 to 4) and the brain region (DLS or DMS) (see the Methods Section for the complete list of tested crossed effects). The main results are shown in Table 2. We found that in general DMS has stronger connections than the DLS (line 6 of the table). Within the DLS and DMS networks, FSIs and MSNs do not have the same role. Indeed, FSIs are mainly the target of the strong connections (line 1) particularly in the DLS compared to DMS (line 8), but this effect disappears in stage 4 (line 7). Conversely, MSNs are the main source of strong connections (line 2) and this effect is stronger in the DLS than in the DMS (line 8). Altogether these results indicate that FSIs may work as a hub receiving strong connections from the surrounding MSNs, and this effect is more evident in the DLS compared to the DMS. In addition, during late learning stages (from 2 to 4), the connections increase their strength (lines 3 to 5) in the DLS but not in the DMS (lines 10 to 12).

**Figure 5.**
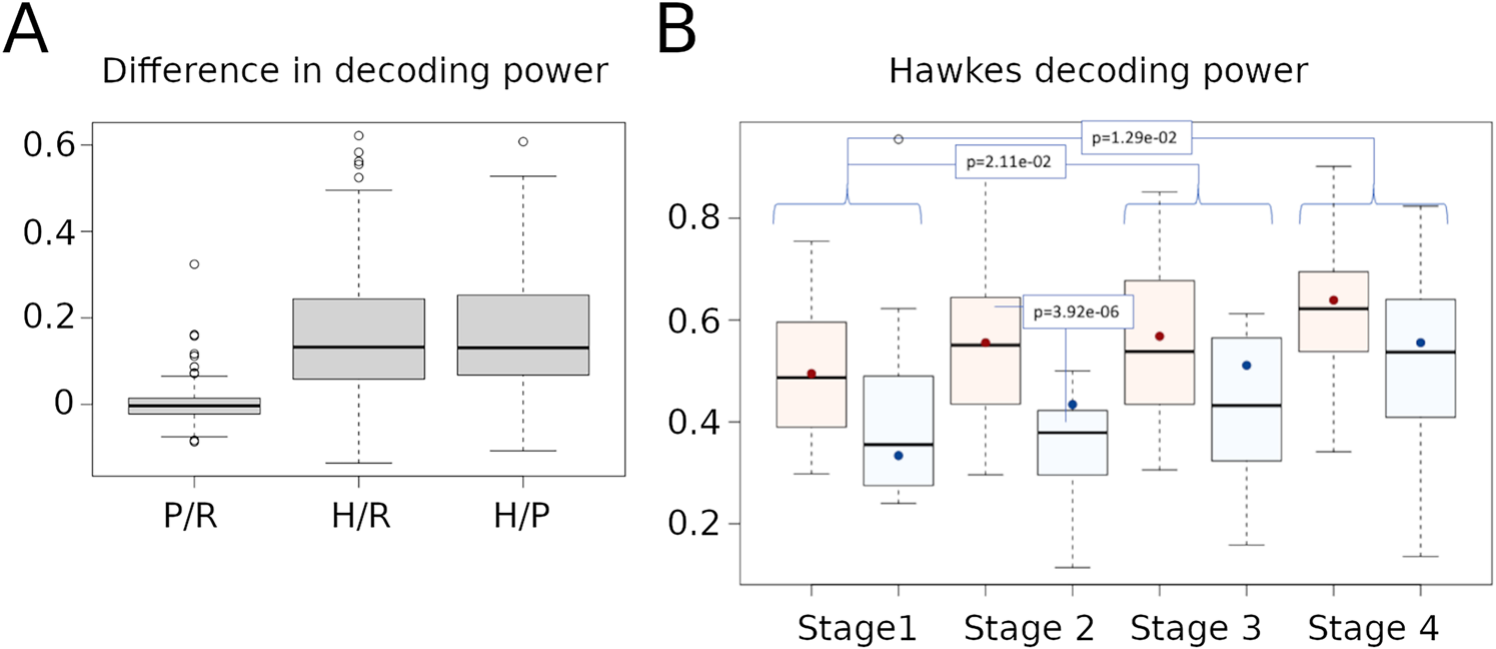
Decoding power. ***A***, Boxplot showing the difference in decoding power (see Methods section for the precise definition) between the random guess (R; corresponding to 1/Nb of Paths), the Poisson model (P) and the Hawkes model (H), for all rats and training sessions. ***B***, Boxplot showing the Hawkes decoding power for DMS and DLS neurons across learning stages (red for DMS and blue for DLS). A beta regression with probit link is performed to compare DMS and DLS across learning stages. All *p*-values have been adjusted for multiplicity with Benjamini-Hochberg method. The colored dots represent the value predicted by the regression model for each boxplot. Significant adjusted *p*-values of the main comparisons are indicated in the figure. Extended data Figure 5-1 shows the Hawkes decoding power of FSI and MSN networks; Extended Data Figure 5-2 shows the decoding power of DLS and DMS “Left-Right Turns” neuron networks across learning stages.

We then used the networks reconstructed with the Poisson and the Hawkes models to decode animal behavior. We quantified the decoding power as the percentage of correct identification of the traveled paths, calculated by estimating the models on 2/3 of the paths in each training session and testing the model predictions on the remaining 1/3. As shown in Figure 5*A*, the Hawkes model has better decoding power than the Poisson model, which predicts rat performance as well as a random guess. This may be due to the short path lengths (see Fig. 1*B*) combined with a fairly low firing rate of the neurons, which results in the prediction being based on 10–20 spikes per neuron in a given session. By taking into account other parameters of neuronal activity (i.e., the direction and strength of neuronal interactions), the Hawkes model is able to decode more efficiently the paths taken by the rats.

10.1523/ENEURO.0436-23.2024.f5-1 Figure 5-1 Boxplot showing the difference in decoding power between the random guess (R; 1/Nb of Paths), the Poisson model (P) and the Hawkes model (H), for all cells (grey), FSI (orange) and MSN (green) networks. Download Figure 5-1, TIF file.

10.1523/ENEURO.0436-23.2024.f5-2 Figure 5-2 Decoding power of ‘Left-Right Turns’ neuron networks. **A**: Boxplot showing the difference in decoding power (see Methods section for the precise definition) between the random guess (R; 1/Nb of Paths), the Poisson model (P) and the Hawkes model (H), for all rats and training sessions. **B**: Boxplot showing the Hawkes decoding power for DMS and DLS neurons across learning stages (red for DMS and blue for DLS). A beta regression with probit link is performed to compare DMS and DLS across learning stages. P-values have been adjusted for multiplicity with Benjamini-Hochberg method. The colored dots represent the value predicted by the regression model for each boxplot. Significant adjusted p-value of the comparaison between stage 1 and 2 is indicated in the figure. Download Figure 5-2, TIF file.

We then compared the decoding power computed by the Hawkes model in DMS and DLS across learning stages (Fig. 5*B*). To do so, we performed a beta regression with probit link (and explanatory variables: “number of paths,” “number of neurons,” “average firing rate,” “DMS-DLS,” “learning stage,” and “DMS-DLS”/“learning stage” cross-effects) to disentangle the effects of interest (“DMS-DLS” and “learning stage”) from other auxiliary effects we need to consider. The results show that, in general, the decoding power in the later learning stages is greater compared to stage 1 (stage 3: adj. *p*-val. = 2.11e–02 ; stage 4: adj. *p*-val. = 1.29e–02). The number of paths (adj. *p*-val. = 4.7e–12) and neurons (adj. *p*-val. = 2.3e–07), as well as their average firing rate (adj. *p*-val. = 1.3e–02) also have a significant effect, but no cross effect was observed. As for the comparison between DMS and DLS, the results show that the decoding power of DMS is globally greater than that of DLS (adj. *p*-val. = 2.8e–03), and particularly during learning stage 2 (adj. *p*-val. = 3.92e–06). Note that the network size effect (number of neurons) has been taken into account in the model and cannot explain such difference. These results show that both the DMS and DLS neural networks undergo a gradual reorganization during learning, since the decoding power of the network increases as the rats performance improves in both DMS and DLS. However, it should be noted that DMS shows greater decoding power relative to the DLS during initial learning, possibly indicating that the acquisition of a spatially guided task rule without any temporal or cue instruction mainly requires DMS neural activity. We estimated the decoding power of the connectivity graphs reconstructed exclusively from FSI and MSN (respectively 86 and 136 sessions). Overall, the results are very similar to those observed for the entire networks. First, the Hawkes model has better decoding power than the Poisson model for both FSI- and MSN-networks (Extended Data Fig. 5-1). Second, DMS decoding power is always greater than DLS, in both networks (FSI, adj. *p*-val. = 1.89e–03; MSN, adj. *p*-val. = 5.46e–03). Finally, the decoding power increases across learning stages (FSI, stage 3: adj. *p*-val. = 5.21e–03, stage 4: adj. *p*-val. =  2.03e–02; MSN, stage 4: 4.92e–02), and this effect is more pronounced for DMS-MSN than DLS-MSN (“DMS-DLS”/“learning-stage” cross effect: adj. *p*-val = 4.92e–02). Note that for FSI we could not test any cross effect, given the low number of sessions analyzed.

Finally, we tested whether the Hawkes model was able to decode animal’s paths as efficiently if the analysis was focused on more restricted population of coding neurons (i.e., “Task-event” and “Left-Right Turns” neurons). The results are nevertheless limited. As for the “Task-event” neurons, it was not possible to reconstruct any graph given the low number of spikes included in each event bin as a consequence of their short duration (task events are based on 100 ms bins and the interactions in the Hawkes model are evaluated in 60 ms time range). As for the “Left-Right Turns” neurons (i.e., neurons activating differently in the central stem depending on future left or right turns), the Hawkes model displayed greater decoding power than the Poisson model, similarly to “Full Paths” neurons (Extended Data Fig. 5-2*A*) but was unable to show any difference between DMS and DLS neurons (Extended Data Fig. 5-2*B*). The decoding power for both DMS and DLS was significantly greater in learning stage 2 (i.e., when animals start to perform correctly) compared to the other stages. This possibly indicates that the information provided by the network activity in the central stem is relevant exclusively for the acquisition of the learning rule but no longer sustains animal performance afterwards.

## Discussion

In this study, we aimed at characterizing how DMS and DLS activation evolves across learning, by analysing how neural activity at the level of single neurons and neural networks modifies in rats learning a continuous spatial alternation task. We showed that DMS and DLS display different task-related activity throughout learning stages. However, the progression of the coding ability across learning does not correlate with the capacity to decode animal performance. At the level of the network both structures increase their decoding power over time. Nevertheless, the DMS displays a greater decoding capacity in all learning stages compared to the DLS, despite a progressively decreasing number of task-coding neurons. Conversely, the smaller coding capacity of the DLS relative to the DMS is paralleled by an increasing number of coding neurons. These results suggest that the DMS and DLS neural networks differently reorganize during learning in order to progressively increase their control over the behavioral performance.

### DMS vs DLS single neuron coding properties across learning stages

We demonstrated that the DMS shows the greatest proportion of coding neurons across all learning stages, possibly indicating that it is more involved than the DLS in learning a spatial alternation strategy. During initial learning, DMS neurons tend to activate and synchronize at the intersection of the maze just before the rat chooses to turn into the left or right arm. Neural synchronization has been related to stimulus encoding in different cortical areas (Grammont and Riehle, 2003; Avital et al., 2013) and can be possibly generalized to the encoding of abstract information such as the learning rule. Therefore, DMS activity at the maze intersection possibly reflects its implication in the acquisition of the spatial rule, or could even sustains deliberative processes (i.e., decision-making). It should be noted that previous studies have ascribed a similar role to the hippocampus and the prefrontal cortex as well as their interaction (Hassabis et al., 2007; Benchenane et al., 2010; Spiers and Gilbert, 2015). Our result supports the hypothesis that DMS could be part of a network mediating decision-making in spatially guided behaviors (Redish, 2016).

In contrast to the DMS, DLS neurons during early learning show mainly activation at action boundaries, as previously described in a cued (i.e., non spatial) T maze (Jog et al., 1999; Thorn et al., 2010) and in a lever-press task (Jin and Costa, 2010). This “bracketing activity” is supposed to improve the acquisition of behavioral routines and promote habit learning. Here, we observe a similar “bracketing” activity in the DLS but in the absence of any specific instruction, thus suggesting that a decomposition of the global task in multiple sub-tasks is at play even in the absence of any clear start-stop signals. This hypothesis is sustained by a recent study in which it has been shown that in the continuous T-maze task rats tend to use a behavioral strategy that consists in chunking sub-actions to perform complete paths (James et al., 2023). Altogether these results point to a role of the DLS in identifying sub-actions or sub-tasks not exclusively in the context of habit learning.

As learning progresses, DMS and DLS single neurons show comparable coding properties, both activating at action boundaries in learning stage 4, similarly to what (Vandaele et al., 2019) reported in a lever-press task. However, despite this progressive similarity, the activity of DMS and DLS neural populations strongly diverge across learning, since the proportion of coding neurons and the entropy of neural ensemble firing follow opposite trends in the two striatal areas. It is thus difficult to draw any clear conclusion based on single neuron firing properties. Therefore we decided to take advantage of a functional connectivity approach based on Hawkes processes to infer neural network functions across learning.

### Decoding rat behavior from DMS/DLS network activities using Hawkes processes

One way to infer brain structure’s functions in learning processes is to decode animal behavior from neural activity. Therefore, instead of looking for the conditions in which neural activity changes, we analyzed how well we can predict animal performance from neural activity. To do so, we used a classical Poisson model based exclusively on single neuron firing rate, and the Hawkes model that takes into account both single neuron firing rate and the interaction between neurons as well as the direction of this interaction. This model allows to reconstruct a graph of functional connectivity between simultaneously recorded neurons, by analysing how much of the activity of a neuron is explained by the activity of the surrounding neurons. We observe that, compared to the Poisson model, the Hawkes model better decodes animal performance, indicating that neural interaction is a strong component of the striatal network function.

Using this analytical approach, we show that despite the progressive decrease in the proportion of coding neurons, the DMS network has higher decoding power than the DLS throughout all learning stages. We observed the same effect on isolated FSI and MSN functional networks. This result supports the hypothesis of a more selective implication of the DMS compared to the DLS in spatial alternation (Moussa et al., 2011). We then assess whether FSIs and MSNs exert a similar control over DMS and DLS networks. Interestingly, these neuron types do not have the same role within the network since FSIs are mainly the target of strong connections particularly in the DLS. FSIs in general provide feed-forward inhibition within the striatum (Mallet et al., 2005), thus shaping striatal network functions by promoting specific neural ensembles that are required for adapted behavior (Owen et al., 2018). Our results suggest that FSIs in freely behaving animals may exert stronger control over striatal network in the DLS compared to the DMS. This hypothesis needs further investigations to be confirmed. In particular, a direct comparison of FSIs activity simultaneously recorded in DMS and DLS would help elucidating this question.

Despite these differences in the functional connectivity, the decoding power of both DMS and DLS increases over training, suggesting that as learning progresses neural activity in both areas conveys information necessary to support behavioral performance. It is interesting to note that the evolution of task-responsive neurons and their decoding power across learning stages are decorrelated. Specifically, the proportion of task-related coding neurons decreases in the DMS but the network itself exhibits enhanced decoding capacity. Conversely, the number of coding neurons and the decoding power increase over time in the DLS. Thus, an increasing proportion of coding neurons not necessarily implies an increased ability to decode animal behavior from the same neural activities. Thanks to the Hawkes processes, we are able to show that a neural network is capable of reorganizing itself to maintain or even increase its decoding power, and hence optimize behavioral performance, with a decreasing number of neurons.

In this context, it is necessary to define which behavior is decoded. Here we consider the decoded performance in terms of elementary paths, as described in Figure 1. We performed the same analysis by taking into account exclusively the central stem of the maze (before the rat turns into the left- or right-goal arm), but no difference was observed between DMS and DLS, and no changes across learning (except for a significantly increased decoding power during initial learning in both structures). It could have been relevant to decode animal behavior based on the networks reconstructed during the task events. Unfortunately, this was not possible since the Hawkes model needs a relatively large number of spikes per neuron in order to reconstruct a functional network. Given the short duration of task event bins with respect to the time range of the interaction function, this condition was not fulfilled. Similarly, it would have been interesting to apply the Hawkes model to the different sub-paths (or sub-actions) that compose each path. Indeed, in a recent study, we have shown that in the same continuous alternation task rats uses a learning strategy that consisted in chunking different sub-actions (James et al., 2023), similarly to what was previously suggested for the acquisition of complex tasks (Botvinick et al., 2009). We have tried to use the Hawkes model on the different sub-actions. However, given the short duration of these sub-paths, the number of spikes per neuron was not sufficient to perform the analysis. In the future, it would be interesting to design behavioral experiments where the sub-actions (or task-relevant events) are long enough to allow to use the Hawkes decoding approach. Alternatively, it would be useful to increase the number of recorded neurons. In that regard, it should be noted that we obtained very good decoding results despite a relative low number of coding neurons recorded per session, which indicates that the striatal network is highly redundant (Reynaud-Bouret et al., 2021).

## Conclusions

In summary, we show that inferring neural network functions based exclusively on task-related single neuron firing activity is insufficient. By using the Hawkes model to decode behavior from neural network activities, we were able to achieve a more comprehensive description of DMS and DLS functions across learning. Altogether our results show that the coding pattern evolves differently in the two striatal areas over training, but both supports learning from early to late stages.

It remains to be determined which is the exact nature of DMS and DLS interactions during learning. In this study, DMS and DLS neurons were not recorded simultaneously, thus preventing the possibility to investigate the dynamic interplay between these two areas across learning. In addition, given the nature of the task that involves lateralized responses, it is possible that more complex interactions exist between the side of the response and the hemisphere where the neurons were recorded (although recording were made in both hemispheres in this study). One possibility is that DMS and DLS activity supports distinct learning strategies and control action selection through a competitive mechanism. The results we obtained at early stages of learning tends to support this hypothesis, but the increased engagement of the two areas across learning stages is not coherent. Alternatively, we may speculate that either DMS- or DLS-mediated action selection process occurs exclusively at the beginning of the learning, or that action selection is operated by the upstream cortical areas, and the differential striatal properties testify (and possibly amplify) such process. Once the behavioral strategy has been implemented, both striatal areas may be implicated in maintain the performance, that may explain why the decoding power in both areas increases over training.

## Synthesis

Reviewing Editor: Michaël Zugaro, CNRS, Collège de France, Inserm Decisions are customarily a result of the Reviewing Editor and the peer reviewers coming together and discussing their recommendations until a consensus is reached. When revisions are invited, a fact-based synthesis statement explaining their decision and outlining what is needed to prepare a revision will be listed below. The following reviewer(s) agreed to reveal their identity: NONE.

The potential interest of this study for the community is twofold. On the one hand, in the context of the role of the different anatomo-functional compartments of the dorsal striatum (DLS and DMS), this study mostly confirms previously published results. The advance is somewhat incremental, but useful for the field. On the other hand, the study uses new tools to analyze and correlate firing activity of striatal output neurons with the different stages of procedural learning - namely the Hawkes process, a method that has rarely been applied to the analysis of neural activity recorded from the brains of behaving animals. Here, the interaction between neurons are represented as directional graphs. This report demonstrates that this method can be used for actual neural activity analysis, and may contribute to the future elucidation of neural information processing mechanisms.

Regarding the striatum, the present work shows MSN &FSI recordings in DMS and/or DLS of behaving rats during a procedural learning in a Y-maze. The authors, thanks to various analysis (including the Hawkes process), show that the proportion of coding striatal neurons decreases in DMS while it increases in DLS across learning, with in both cases an increase of the decoding power. Overall, this is in agreement with previous studies.

Major comments:

1. It seems important to show that this new analytical method has been used successfully. However, perhaps due to insufficient understanding of the method on our part, the degree of application seems to be insufficient. In the early half of this report, detailed analyses were performed to determine the characteristics of neurons, such as which paths and where in the paths the neurons were active. However, the Hawkes model analysis does not mention many of the details of such neuronal characteristics. For example, each neuron showed high frequency activity at any different task event like turning. Would the results of the Hawkes analysis differ between groups of cells with overlapping active times and those without such overlapping times? Also, is it not possible to perform Hawkes analysis for each Task event? or is it because of the limitations of the Hawkes model analysis? Even if there are limitations, it might be a significant report if the limitations of this method are reported and discussed in detail and a roadmap for future use is provided.

2. Abstract/Introduction/Discussion. The authors chose a rather black and white (and 'old') view regarding the engagement of DLS and DMS during learning: DMS then DLS during early and late stages, respectively. This 'classical' view has clearly been challenged in recent years, and a significant number of studies reported that part of DLS is already engaged (in terms of firing activity or plasticity expression) from the early stages concomittantly with DMS. The authors should cite these studies in the Introduction/Discussion and moderate the 'classical' model in the Abstract. As an example there is no "common assumption of a gradual shift from DMS to DLS activity".

3. Results, page 9. "since no difference was observed between MSN and FSI, both were included in the analysis". Nevertheless, it will be useful to see these two populations analyzed separately on different graphs. Please provide such analysis and illustrations.

4. Experiments were conducted in 8 rats, mainly recorded either in the DLS or DMS. Please add a 'limitation' chapter in the Discussion since it is difficult to firmly conclude with populations of neurons recorded in different animals and learning stages and then build connections and decoding power accross them. For example, the authors state in the Discussion "In this study, we aimed at characterizing the dynamic interplay between DMS and DLS accross learning" which is almost impossible with unilateral recordings in either DLS or DMS. Also in the limitation: this Y-maze task is a lateralized task, and because the DLS and DMS recordings were performed in the right or left hemisphere, interpretations could turn out to be highly complex. Please add few sentences on that.

5. Figure 4. It will be very interresting (and required) to see a the session 11 for DLS and the session 6 for DMS, for comparison (with session 11 for DMS and 6 for DLS).

6. Since the authors recorded almost as many FSIs as MSNs, they should provide more information regarding the quality control of spike sorting, showing a few autocorrelograms and recordings of FSIs vs MSNs.

Minor comments:

1. Figure 1b. Is it a representative curve? averages from n rats? could you please better explain this panel?

2. Results, page 5. "We found 65.5% of ..."; it is not clear at which stages of the training these results have been obtained. Also these "Coding neurons" are MSNs + FSIs or just MSNs?

## Author Response

We thank the reviewers for all the time they spent on this manuscript and their constructive comments. We have made all changes they asked. The responses to all raised issues are reported below. The modified text in the manuscripts is highlighted in red. Responses to reviewers Major issue : Please provide the results of the decoding analyses using MSN and FSI populations separately. R : We analyzed the decoding power of FSI and MSN networks separately (respectively 86 and 136 sessions). Overall, we confirmed the results observed using the entire networks. The results are reported in the result section page 17 and a figure was added in the supplementary material (Extended Data Figure 5-1). We showed that the Hawkes model has better decoding power than the Poisson model for both FSI and MSN networks, and DMS decoding power is always greater than DLS. Finally, the decoding power increases across learning stages, and this effect is more pronounced for DMS-MSN than DLS-MSN. For FSI we could not test any cross-effect since the number of recording session analyzed per region across learning stages was too low. Minor issues 1. Please document the general criteria used to exclude certain sessions: "We did not provide session 11 for the same DLS rat because it included only one coding neuron (this session was excluded)." R : All sessions with less than 2 'Full path coding' neurons were excluded. In addition, the rat has to perform at least two different paths (among the ten paths described in figure 1), and each path needs to be repeated three times. This information is added page 9. Session 11 was excluded because it included only 1 coding neuron. In total we analyzed 171 out of 206 sessions. This paragraph is included in the method section page 9. 2. Figures (including legends) are not reader friendly. For instance, the texts in figures are small, and there is a strange diagonal line above the word "Correct" in Figure 1C. Please double check all figures and legends. R : All figures were checked and the text size increased. Some legends were modified, we hope that they are cleared than before. 3. The equation for the least-square contrast seems incorrect. Equation (2) in Lambert et al. (2018) computes the distance between the parameter (lambda here, beta in Lambert) and an estimation based on the data (dN in Lambert), but the equation in the current manuscript only includes lambda. R : The least-square contrast defined in our article is exactly the same as in Lambert et al.2018. It is just a different way of writing it. If we take beta as the vector with parameters: μ_*n,k*_ and *a*_*m,n,i*_, then Λ_*n,k*_ can be rewritten as : Λ_*n,k*_(*t*)=*c*_*t*_^†^ β, with *c*_*t*_ defined as in Lambert et al (2018).
