# Supplementary figures and images for: Coding Dynamics of the Striatal Networks During Learning
Source: eNeuro. 2024 Oct 23;11(10):ENEURO.0436-23.2024. doi: 10.1523/ENEURO.0436-23.2024 (PMC11521795; doi:10.1523/ENEURO.0436-23.2024)

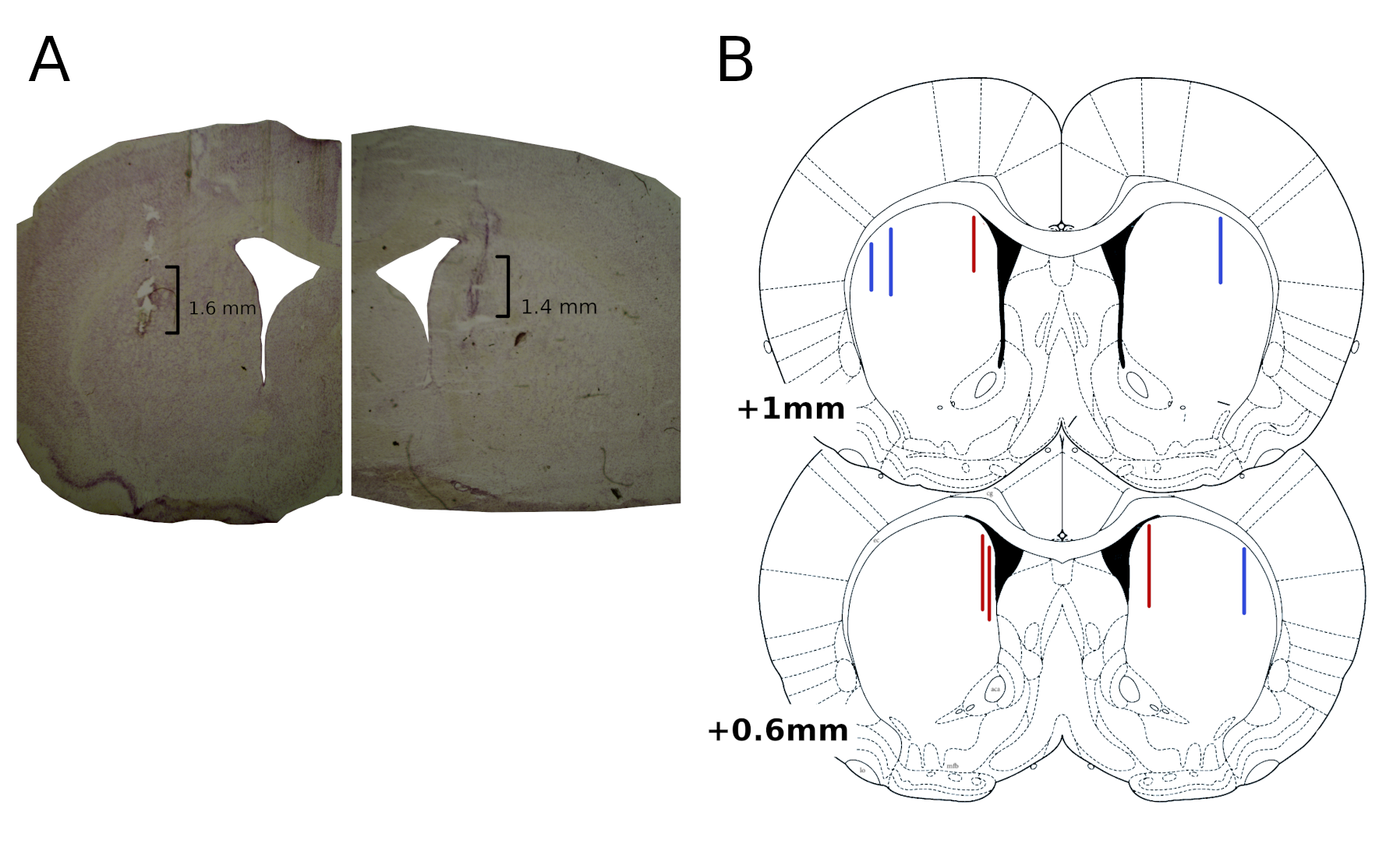

Supplement: Figure 1-1 — Reproduction of tetrode positions and recording sites in the DMS and DLS. A: Photos of Nissl-stained coronal sections from two representative rats implanted in the DLS (left panel) and DMS (right panel). The tetrode tracks are visible in both sections. The vertical black bars represent the lengths of striatal tissu from which neural recordings were performed. B: Representation of the recording sites from all rats (DLS in blue and DMS in red). Download Figure1-1, TIF file. [file eneuro-11-ENEURO.0436-23.2024-s002.tif]

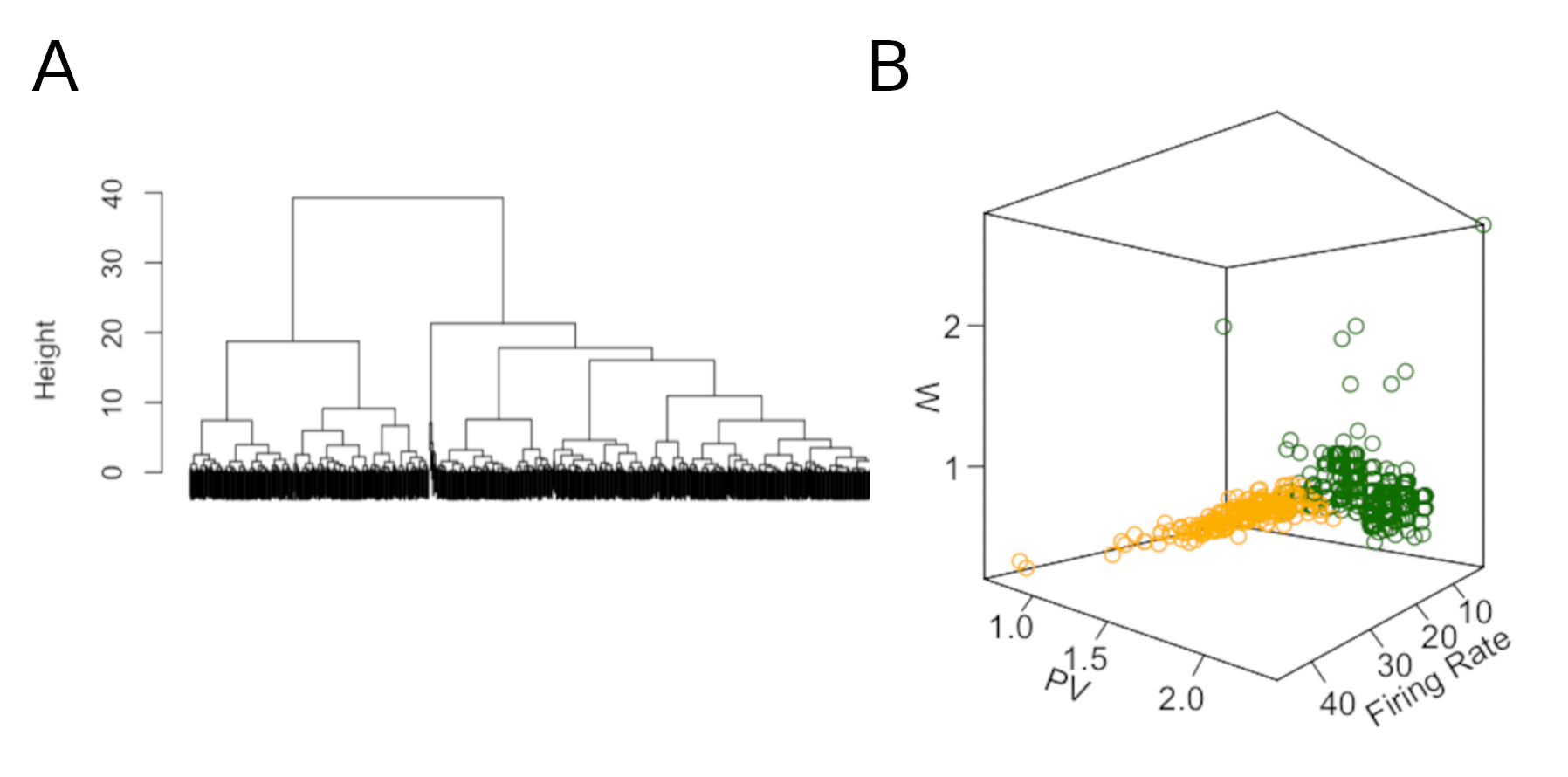

Supplement: Figure 1-2 — FSI-MSN classification. A: Dendogram of the hierarchical clustering (method “Ward D2”) of neurons based on waveform (wf) properties (Peak-Valley distance (PV), Width at mid-height (W)) and firing rate. Two clusters are identified using K-means method (with K = 2). B: 3D representation of wf properties and firing rate of the two clusters. Fast Spiking Interneurons (FSI) and Medium Spiny Neurons (MSN) are represented in orange and green, respectively. A total of 451 MSN and 199 FSIs have been identified. No significant difference in the proportion of FSI/MSN was observed between DMS and DLS (p-value = 0.97). Download Figure 1-2, TIF file. [file eneuro-11-ENEURO.0436-23.2024-s003.tif]

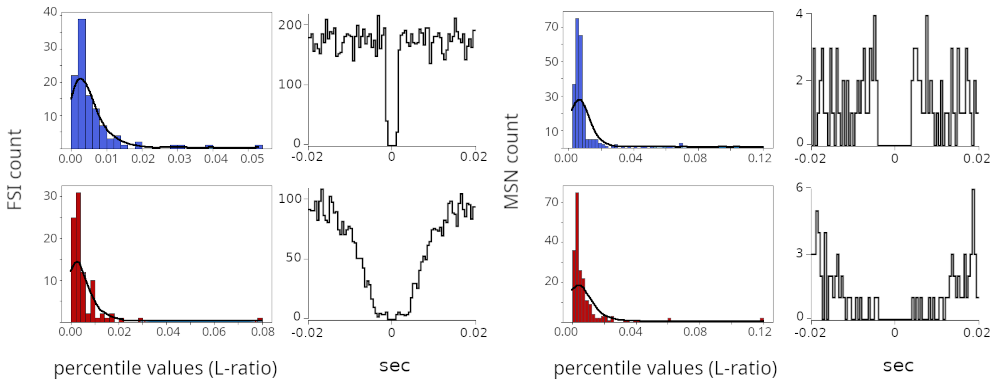

Supplement: Figure 1-3 — Estimation of spike sorting quality for FSIs and MSNs. From left to right: distributions of the percentile values of L-ratio measures (with k-means density curves in black) for all FSIs (left) and MSNs (right) recorded from DLS (in blue) and DMS (in red); examples of spike clusters and spike autocorrelograms from 2 neurons for each category (FSI - DLS and DMS, MSN - DLS and DMS). Autocorrelograms were constructed using 20 ms windows and 0.5 ms bins. Download Figure 1-3, TIF file. [file eneuro-11-ENEURO.0436-23.2024-s004.tif]

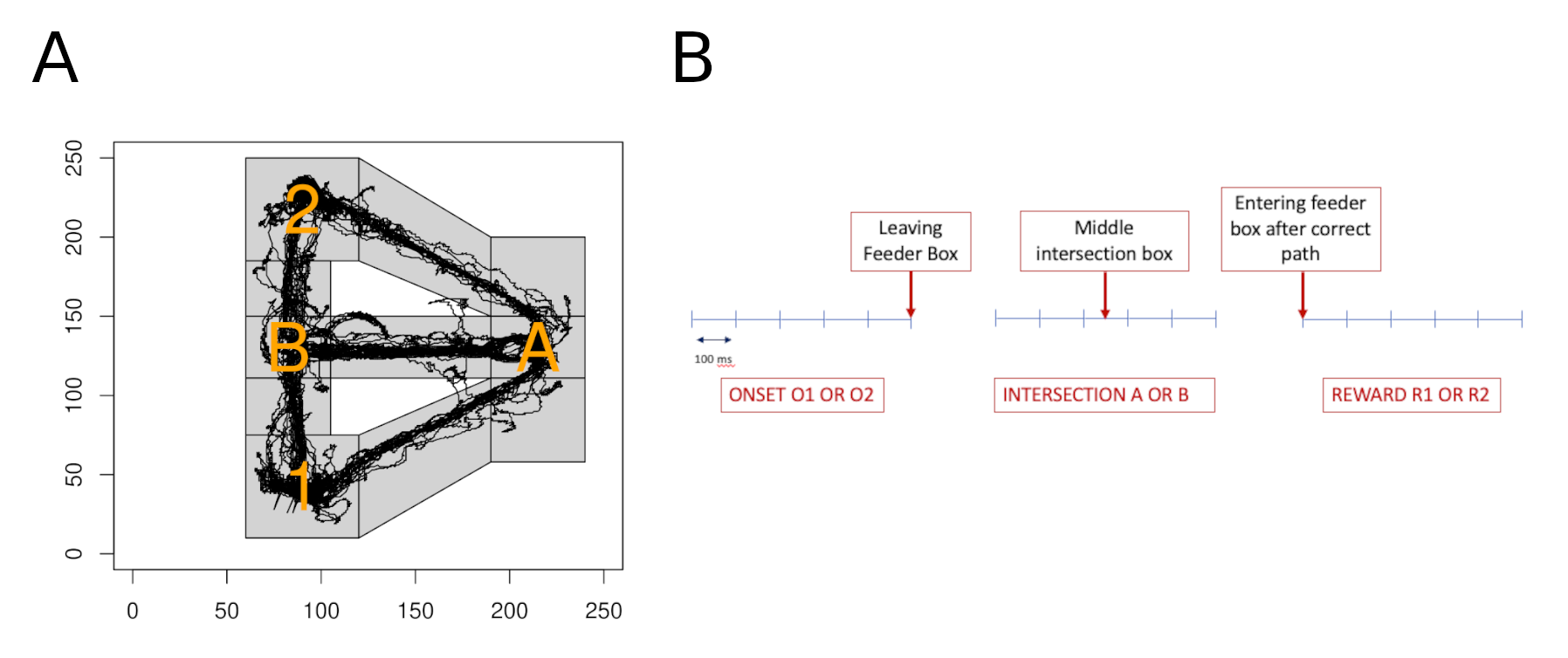

Supplement: Figure 2-1 — Timeline of a typical training session. A: The maze has been divided in boxes. The black line represents the trajectory of the animal during one example session. The feeders are located in the boxes 1 and 2. Maze intersections are located in boxes A and B. B: Each 500 ms task event (centered on the onsets, intersections and reward locations) was divided in five 100 ms bins. This leads to a total of 6 * 5 = 30 time bins. Download Figure 2-1, TIF file. [file eneuro-11-ENEURO.0436-23.2024-s005.tif]

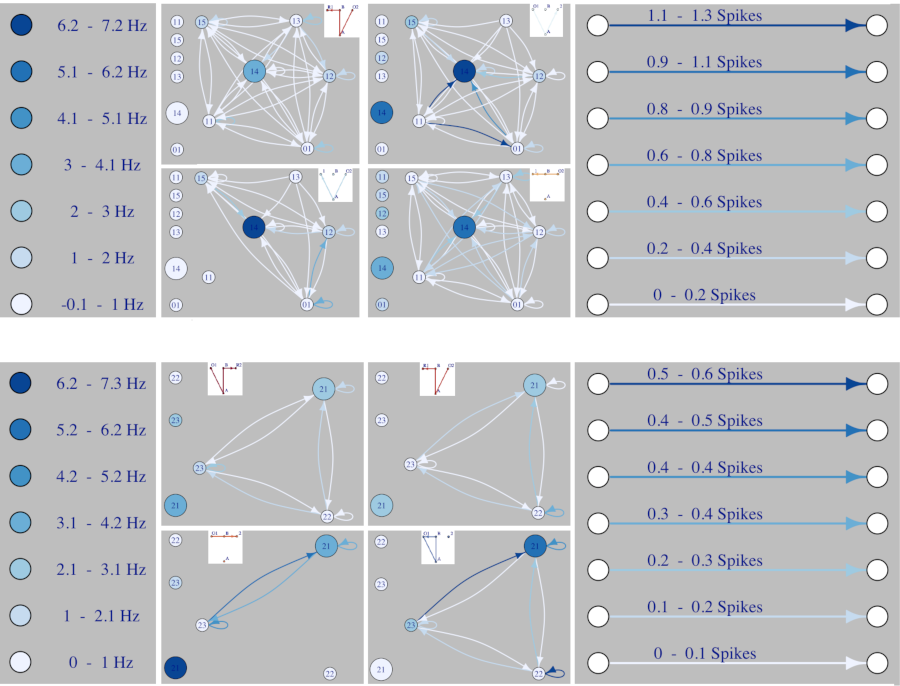

Supplement: Figure 4-1 — Examples of estimated network models for DLS ‘Full path’ neurons from two different rats and two different learning sessions. Upper panel: session 5 - learning stage 2. Lower panel: session 36 - learning stage 4. From left to right: color codes for the average firing rate ; 4 network models of the same neurons reconstructed for 4 different paths (Poisson model on the left, Hawkes model on the right); color codes for the interaction function. Download Figure 4-1, TIF file. [file eneuro-11-ENEURO.0436-23.2024-s006.tif]

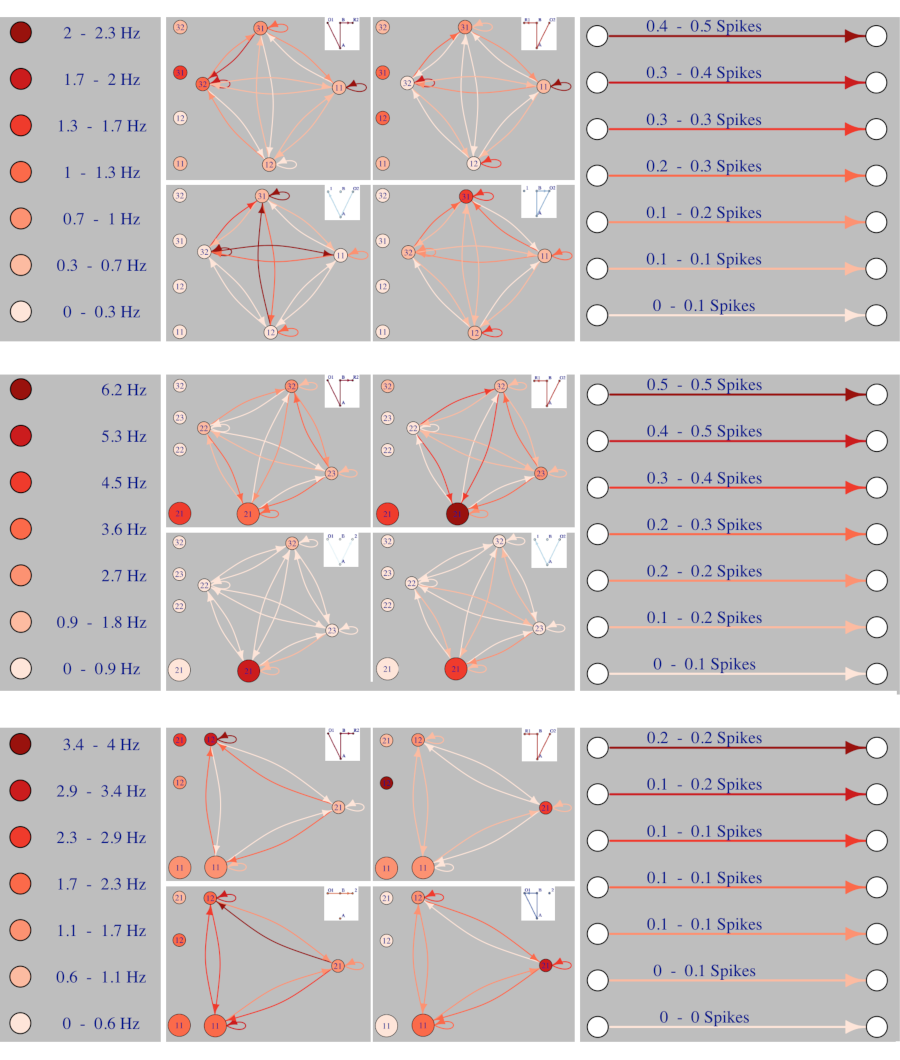

Supplement: Figure 4-2 — Examples of estimated network models for DMS ‘ Full path’ neurons from three different rats and three different learning sessions. Upper panel: session 2 - learning stage 1. Middle panel: session 19 - learning stage 3. Lower panel: session 30 - learning stage 4. From left to right: color codes for the average firing rate ; 4 network models of the same neurons reconstructed for 4 different paths (Poisson model on the left, Hawkes model on the right); color codes for the interaction function. Download Figure 4-2, TIF file. [file eneuro-11-ENEURO.0436-23.2024-s007.tif]

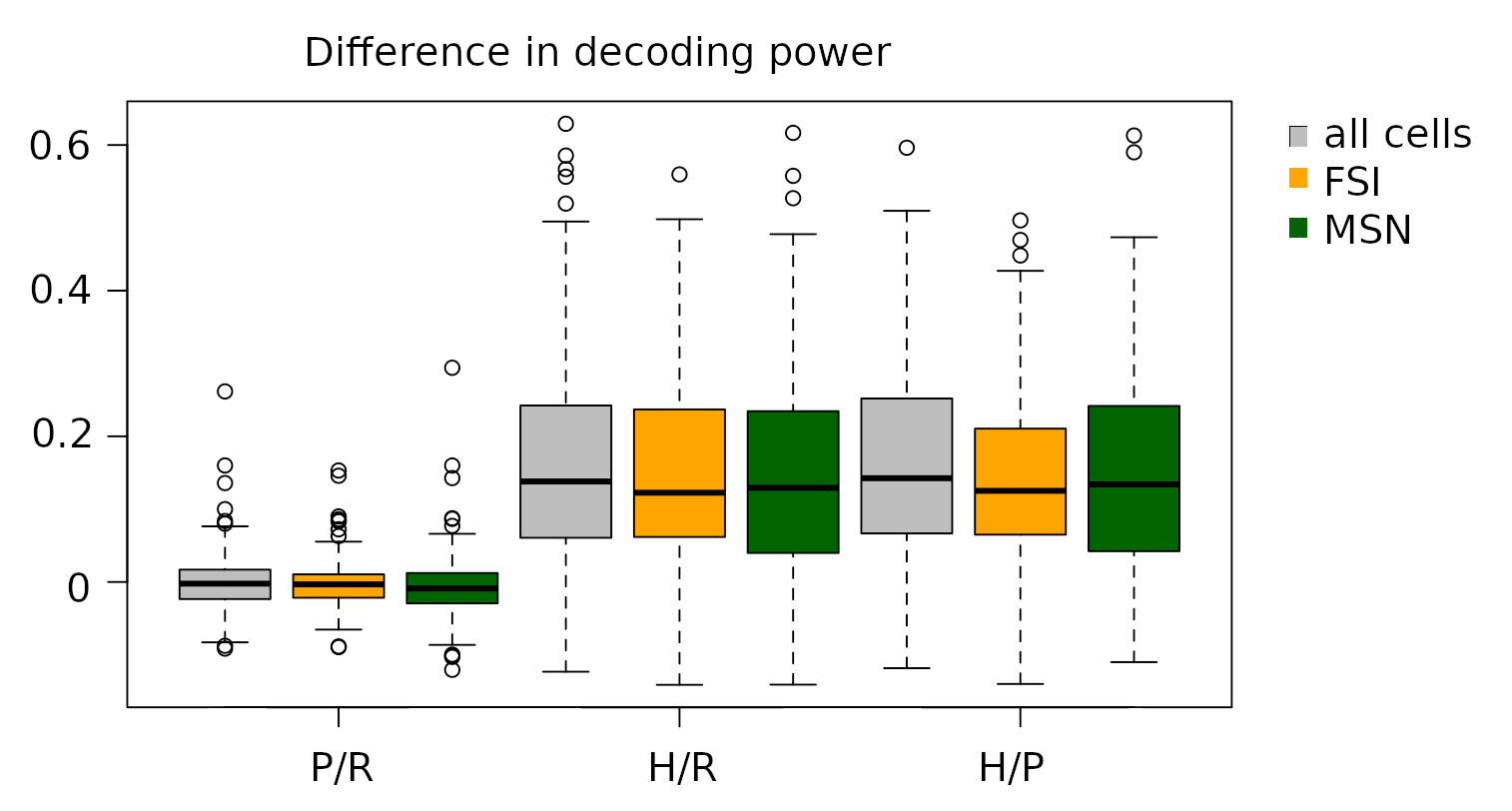

Supplement: Figure 5-1 — Boxplot showing the difference in decoding power between the random guess (R; 1/Nb of Paths), the Poisson model (P) and the Hawkes model (H), for all cells (grey), FSI (orange) and MSN (green) networks. Download Figure 5-1, TIF file. [file eneuro-11-ENEURO.0436-23.2024-s008.tif]

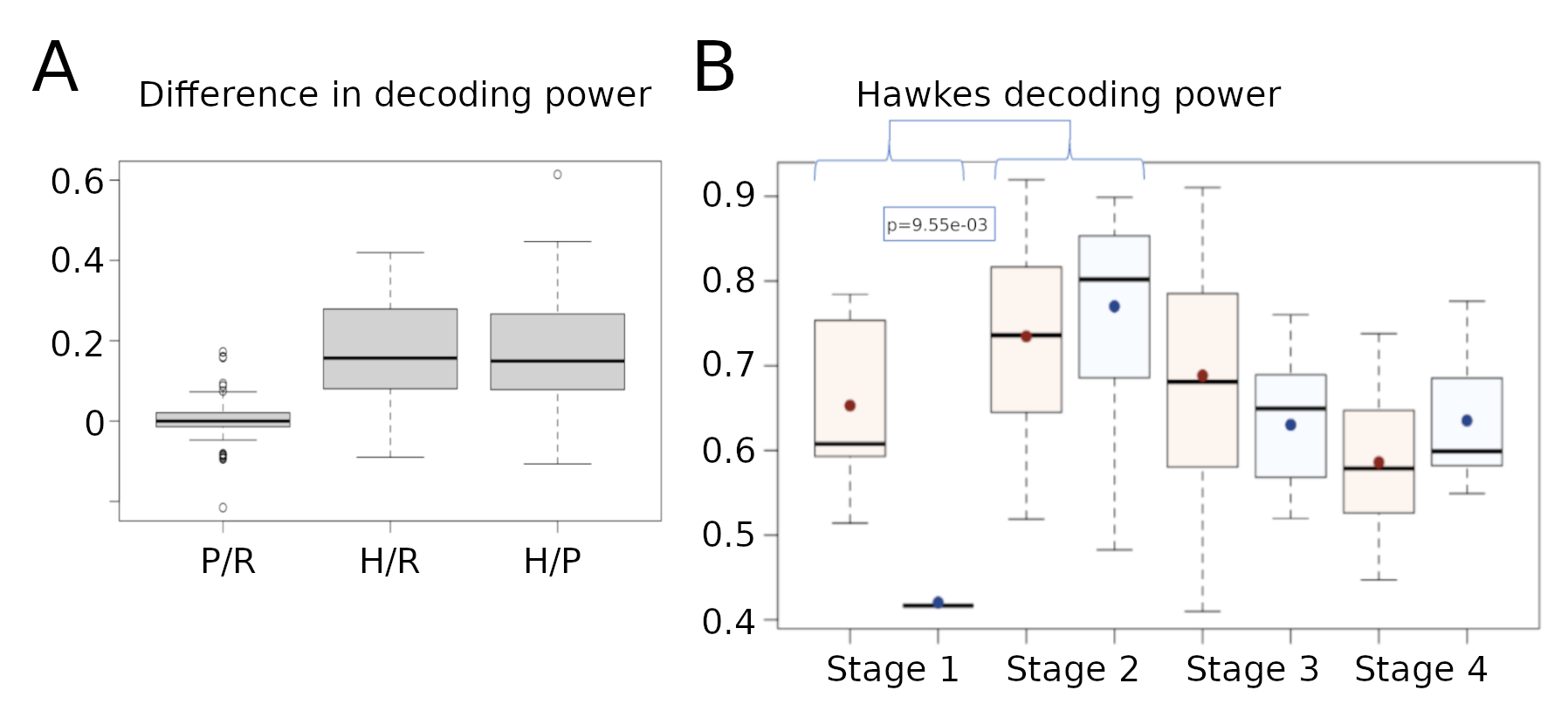

Supplement: Figure 5-2 — Decoding power of ‘Left-Right Turns’ neuron networks. A: Boxplot showing the difference in decoding power (see Methods section for the precise definition) between the random guess (R; 1/Nb of Paths), the Poisson model (P) and the Hawkes model (H), for all rats and training sessions. B: Boxplot showing the Hawkes decoding power for DMS and DLS neurons across learning stages (red for DMS and blue for DLS). A beta regression with probit link is performed to compare DMS and DLS across learning stages. P-values have been adjusted for multiplicity with Benjamini-Hochberg method. The colored dots represent the value predicted by the regression model for each boxplot. Significant adjusted p-value of the comparaison between stage 1 and 2 is indicated in the figure. Download Figure 5-2, TIF file. [file eneuro-11-ENEURO.0436-23.2024-s009.tif]
